# Scalable hybrid framework for real time and non real time task scheduling in fog computing using federated reinforcement learning and PSO GA

**DOI:** 10.1038/s41598-025-22218-5

**Published:** 2025-11-03

**Authors:** Fei Liu, ZhiLi Liu, XiaoHong Liu, Hua Zhou

**Affiliations:** 1 School of Computer Science and Engineering, Hunan University of Information Technology, Changsha Hunan, 410151 China; 2 School of Information and Mechanical and Electrical Engineering, Hunan International Economics University, Changsha Hunan, 410205 China

**Keywords:** Fog computing, Task scheduling, FRL, PSO-GA, Distributed clustering, VAE, Scalability, Load prediction, Latency, MDP, FRAHTOS, Engineering, Mathematics and computing

## Abstract

Fog computing offers a decentralized paradigm to address the low-latency and energy-efficiency requirements of emerging IoT applications. However, the heterogeneity of edge nodes, the dynamic nature of workloads, and the dual need for both real-time and non-real-time scheduling introduce significant challenges in task allocation. This paper presents FRAHTOS, a Federated Reinforcement Learning and Hybrid Optimization Scheduling framework, to address these issues. FRAHTOS integrates Markov Decision Process (MDP) modeling, Federated Reinforcement Learning (FRL) for real-time tasks, and a PSO-GA hybrid optimization algorithm for non-real-time scheduling. Feature preprocessing and dimensionality reduction are performed using Adaptive Variational Autoencoders (VAE), followed by clustering with GMM and DBSCAN, and lightweight labeling using decision trees. The framework further enhances system responsiveness with EDF scheduling and VARIMA-based load forecasting. Simulation results using iFogSim demonstrate 85–95% utility, 86–96% task completion, and 3-5.5 ms latency, outperforming conventional methods. Additionally, the system sustains energy consumption between 50 and 80 mJ, suitable for battery-constrained nodes. FRAHTOS delivers a robust, scalable, and adaptive solution for intelligent IoT task scheduling. Future work includes validation on real-world data and integration with advanced federated simulation platforms.

## Introduction

Fog computing has emerged as a vital paradigm for processing Internet of Things (IoT) data, addressing the fundamental needs for low latency, energy efficiency, and scalability in distributed systems^1,2^. Fog computing, introduced by Cisco in 2012, augments cloud capabilities at the network edge, diminishing reliance on remote servers and significantly decreasing communication latency^3^. The rapid proliferation of IoT devices in sectors including healthcare, smart cities, and autonomous systems has created an urgent need for localized and intelligent data processing^4^. In these circumstances, fog computing facilitates both real-time and non-real-time applications, guaranteeing dependable, economical, and ecologically sustainable operations^5^.

Notwithstanding these benefits, task allocation in fog computing continues to pose challenges owing to node heterogeneity^6^, fluctuating workloads, and constrained resources^7^. Edge devices exhibit considerable variation in computing capability, memory, and connection, whilst fluctuating task arrivals and node mobility contribute to instability^8^. Meeting the rigorous sub-5 millisecond latency requirements of time-sensitive IoT applications, such as real-time medical warnings or autonomous car navigation, while ensuring scalability and data privacy, is a significant challenge. Frameworks for Service Function Chain orchestration in NFV networks optimize task placement and routing to reduce latency and costs for delay-sensitive applications^9^. Similarly, proactive scheduling of concurrent virtual machine migrations minimizes downtime and bandwidth requirements while maintaining task interdependence^10^. In Mobile Edge Computing (MEC), predictive service pre-deployment reduces cold-start latency and handover overheads^11^. These methods highlight the complex equilibrium necessary to get ultra-low latency while ensuring economical, scalable, and privacy-aware resource management in fog computing systems.

Current methodologies elucidate these difficulties more distinctly^12^. Conventional heuristics are ineffective in dynamic and heterogeneous environments, resulting in uneven load allocation and limited scalability^13^.Numerous Deep Reinforcement Learning methodologies (e.g., DQN, DDPG, SAC) enhance decision-making quality but necessitate substantial computational resources, exhibiting sluggish and frequently unstable convergence in resource-limited settings^14^. Similarly, federated frameworks like HAFedRL or PFR-OA tackle privacy and scalability issues but entail significant computing complexity and generally overlook clustering^15,16^. These essential challenges heterogeneity, dynamic workloads, scalability constraints, and privacy remain inadequately addressed, highlighting the necessity for a holistic resolution^17^.

**Fig. 1:**
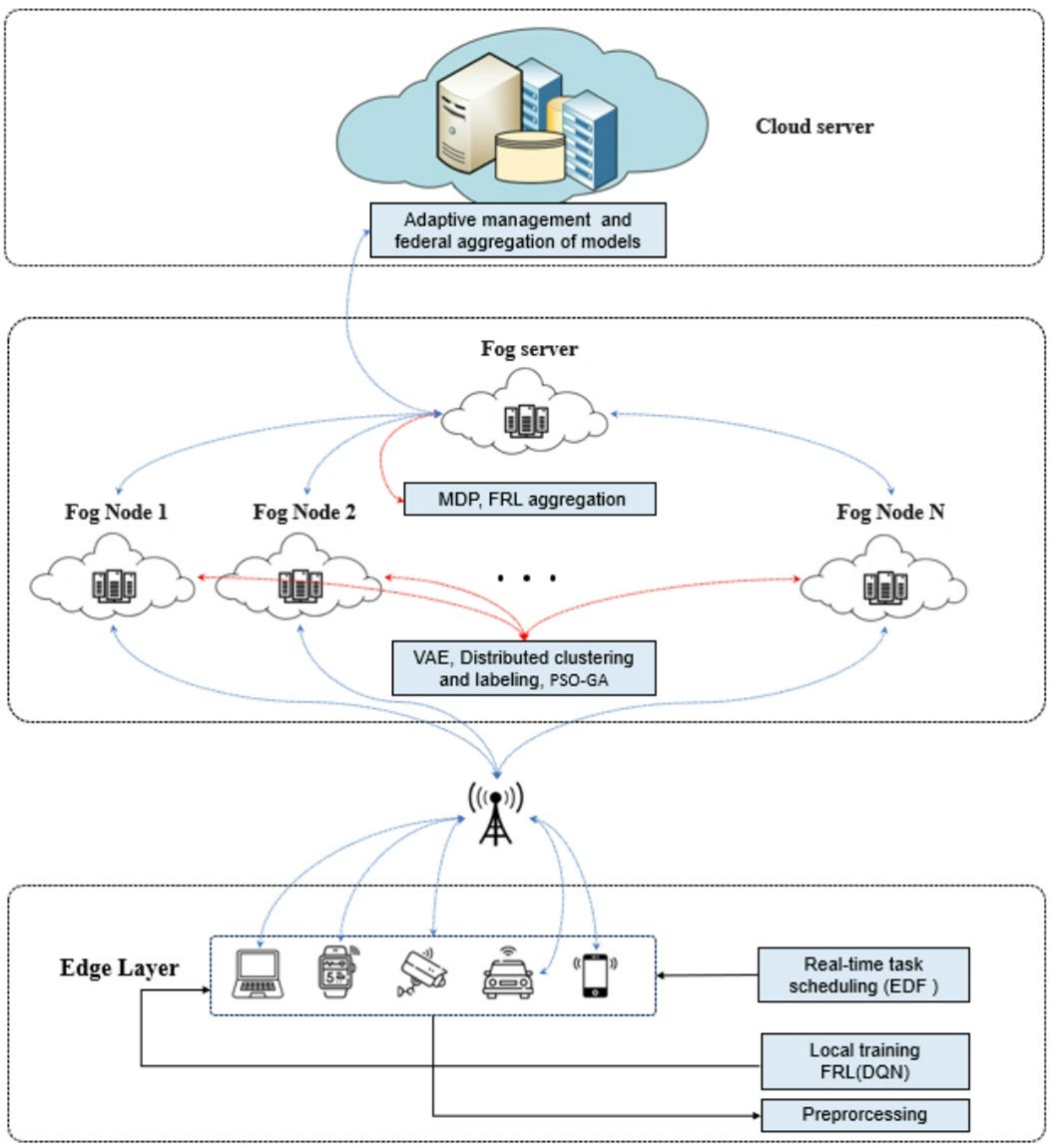
Real-time and non-real-time task scheduling in edge-fog-cloud environments. The layered design of the proposed FRAHTOS framework incorporates preprocessing (VAE), clustering (GMM/DBSCAN), real-time scheduling (FRL), and non-real-time optimization (PSO–GA).

To address these challenges, this study proposes FRAHTOS, a scalable hybrid framework for real-time and non-real-time task scheduling in fog computing. FRAHTOS integrates FRL for real-time scheduling with a hybrid PSO-GA optimizer for non-real-time tasks, supported by VAE/GMM-based clustering for task classification and MDP modeling for dynamic decision-making. Additional innovations include VAE-based feature compression, dynamic caching, and VARIMA load forecasting, which together reduce complexity and enhance energy efficiency. Figure 1 illustrates the general architecture of FRAHTOS, highlighting the interplay between preprocessing, clustering, scheduling, and forecasting components.

For clarity, several technical terms are briefly explained. FRL allows multiple edge or fog nodes to collaboratively train models without sharing raw data, thereby preserving privacy and reducing communication overhead. PSO-GA refers to a hybrid optimization strategy that combines Particle Swarm Optimization (PSO), known for its fast convergence, with Genetic Algorithms (GA), which provide exploration diversity.

**Fig. 2:**
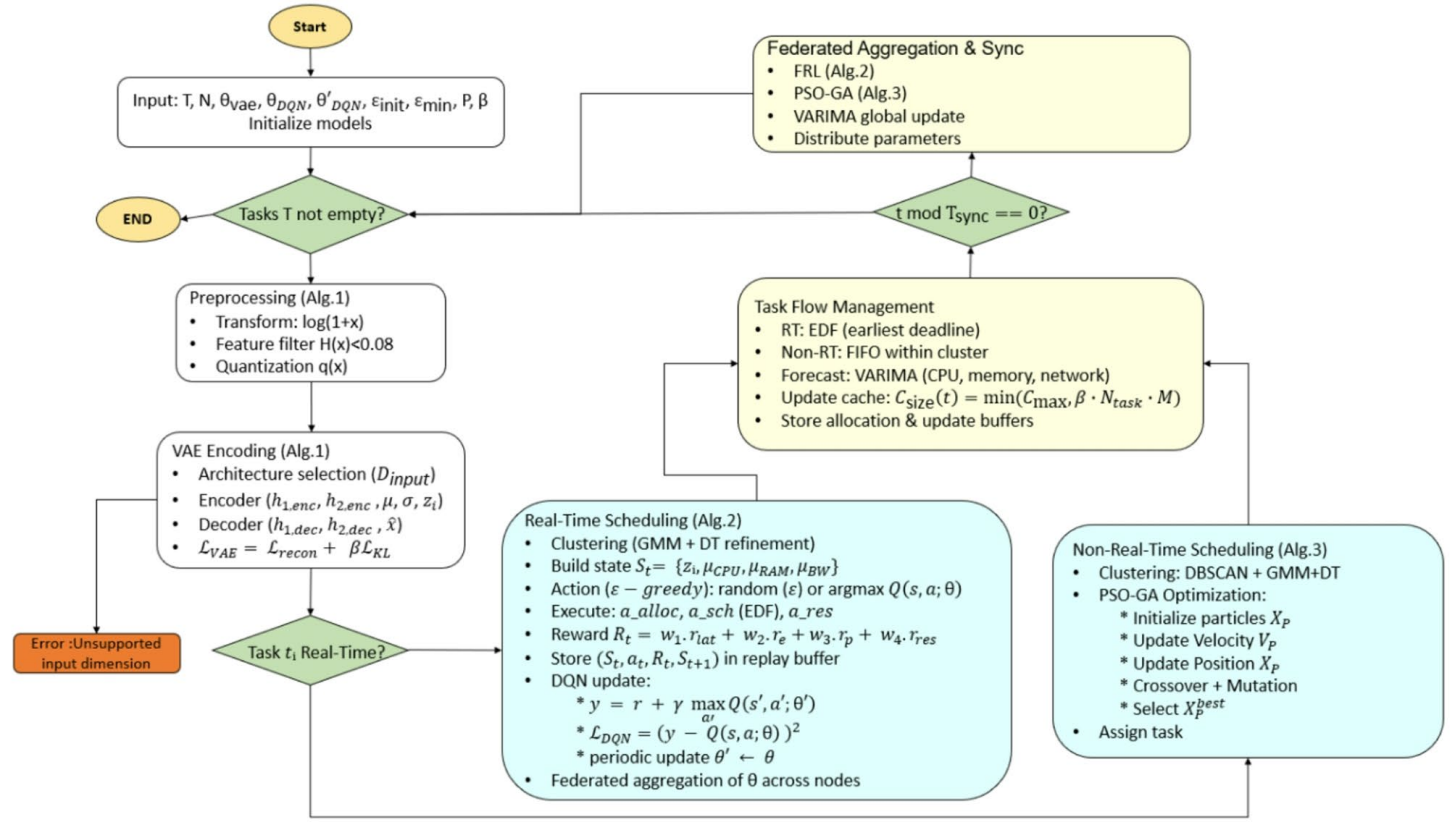
Systematic flowchart of the FRAHTOS framework showing interactions among preprocessing, clustering, FRL, PSO-GA, and final scheduling components.

Figure 2 delineates the comprehensive workflow of the proposed FRAHTOS architecture systematically. Input IoT task and node data are first preprocessed and compressed using VAE, lowering communication and computational overhead. These compressed features are then clustered using GMM, DBSCAN, and lightweight decision trees, which categorize tasks into groups with similar characteristics. The framework then diverges into two modules: FRL for latency-sensitive, real-time tasks and PSO-GA for computationally intensive but delay-tolerant tasks. Finally, the outputs of both modules are combined through EDF-based scheduling and VARIMA forecasting, which enhance workload stability and overall efficiency. This pipeline reflects a holistic design rather than a piecemeal integration.

FRAHTOS is not simply a combination of FRL and PSO-GA; it is a complex architecture designed to address the persistent shortcomings of current methods. For example, FMADRL used SAC to accommodate variable conditions, but faced challenges such as unstable convergence and significant computational demands in large federations^18^. HAFedRL, which combined DDPG with hierarchical programming, increased scalability but imposed excessive complexity on resource-constrained nodes^19^. Algorithms such as DDPG and SAC involve complex state-action relationships. However, they require significant training data and long convergence times, making them unsuitable for latency-sensitive IoT applications.

To enhance understanding for those outside the field of machine learning, this study briefly explains some of the technical terms used in this paper. FRL is a type of reinforcement learning in which many edge or fog nodes collectively train their models while preserving data privacy and minimizing communication costs. PSO-GA represents a hybrid optimization method that combines PSO, which simulates the social dynamics of flocks of birds to identify optimal solutions, with GA, which use biologically inspired mechanisms such as selection, crossover, and mutation. The integration of PSO and GA algorithms increases the convergence speed and solution diversity. VAEs are neural architectures that compress high-dimensional data into lower-dimensional latent representations, increasing the efficiency of subsequent processing. Gaussian mixture models (GMMs) are statistical clustering techniques that group tasks or data points based on probability distributions, facilitating flexible and adaptive classification.

This paper presents the subsequent principal contributions:

- Unified Task Allocation Framework: The amalgamation of FRL, PSO–GA, VAE/GMM clustering, and MDP modeling into a comprehensive scheduling solution that addresses heterogeneity and scalability issues.
- Real-Time Optimization: Implementation of FRL utilizing dynamic clustering and EDF scheduling to attain ultra-low latency (3–5.5.5 ms), essential for medical IoT and autonomous driving.
- Energy-Efficient Resource Management: The implementation of VAE compression and VARIMA forecasting decreases energy usage (50–80 mJ), hence promoting sustainability in resource-limited nodes.
- Scalable Distributed Clustering: The implementation of VAE/GMM clustering combined with decision-tree labeling enhances accuracy and scalability in extensive networks.

The subsequent sections of this work are structured as follows. Section “Related Works” examines pertinent literature on task allocation in fog computing. Section “The structure model and formulation of the problem” delineates the FRAHTOS framework along with its methodological constituents. Section “Experiments and results” assesses FRAHTOS in comparison to baseline approaches with iFogSim simulations. Section “Conclusion” finishes with principal findings and recommendations for subsequent research.

## Related works

Recent work around edge intelligence and large-scale decision systems spans perception pipelines, optimization for operations, and networking/control each illuminating part of the fog scheduling problem yet leaving key gaps in convergence stability, overhead, and adaptability. Vision-centric studies such as joint scene-flow and moving-object segmentation on LiDAR^20^ and image-transformation for defect identification^21^ demonstrate the maturity of deep models at the edge, while multimodal analytics for pilot situation awareness^22^ further evidences the feasibility of complex inference near data sources. However, these lines focus on perception, not compute/task scheduling under heterogeneous resources, and thus do not address how to stabilize learning-based allocators, reduce communication cost, or adapt to workload shifts in fog settings.

Closer to sequential decision-making, reinforcement learning has been used to regulate dynamics in complex networks: RL-driven interventions for emotion contagion^23^ show that policies can remain effective under non-stationary diffusion processes. In production-like scheduling, DQN has been applied to dynamic job-shop with AGVs^24^, evidencing RL’s ability to outperform heuristics when queues, travel times, and machine states evolve rapidly. At the same time, hybrid learning optimization ideas have reappeared in operations research, e.g., knowledge- and data-driven lot-streaming in hybrid flowshops^25^, as well as multi-objective fuzzy optimization for flight scheduling^26^; these works underscore the value of hybridization and explicit trade-off handling but typically lack federated training, representation compression, and cluster-aware adaptation that are crucial for bandwidth/compute-limited fog. Energy-system optimization^27^ likewise addresses large-scale resource coordination, yet the objective, dynamics, and constraints differ markedly from IoT task allocation.

Networking-focused research further tightens the connection to scalability and heterogeneity. Multi-agent RL for scalable routing^28^ targets convergence and coordination across many agents on large graphs, offering insights into stabilizing distributed learning under scale. Meanwhile, task offloading and resource allocation for satellite–terrestrial integrated networks^29^ tackles extreme heterogeneity and tight link budgets an architectural cousin of edge–fog–cloud where communication constraints and topology strongly shape feasible policies. Still, these works generally optimize network-layer routing or offloading strategies and do not co-design the representation, clustering, and dual-track scheduling (real-time vs. non-real-time) required by fog compute orchestration.

Positioning and distinction. Building on the above, FRAHTOS advances beyond a “simple hybrid” in three concrete ways aligned with the reviewer’s criteria:

- Convergence behavior. Unlike centralized or raw-feature RL used in^24^ and the perception-centric pipelines^20–22^, FRAHTOS employs Adaptive VAE to learn compact, smooth latent features before policy learning, and it applies GMM/DBSCAN clustering to structure task types. This cluster-aware, compressed state improves policy landscape smoothness and accelerates/stabilizes convergence, echoing the distributed-coordination lessons from^28^ but at the compute-scheduling (not routing) layer.
- Overhead reduction. Prior work rarely minimizes both computational and communication costs together: RL schedulers in^24^ lack federated updates and transmit/compute on high-dimensional inputs; hybrid OR models in^25–27^ do not address model-update traffic. FRAHTOS reduces overhead through (i) latent compression (smaller models, cheaper inference), (ii) federated averaging (no raw data sharing), and (iii) cluster-conditioned search that narrows the candidate set for the NRT optimizer an approach that is complementary to offloading designs under harsh links in^29^.
- Adaptability to highly dynamic, heterogeneous fog. While^24–26^ demonstrate adaptability in manufacturing/transport settings, they do not separate latency-critical from compute-intensive tasks or react with different solvers. FRAHTOS uses a dual-track architecture: FRL + EDF for real-time deadlines and PSO–GA for heavy, non-real-time batches. Combined with VARIMA forecasting and dynamic caching, this yields responsiveness to fast queue/load changes (akin in spirit to MA-RL responsiveness in^28^ and robustness to long-horizon workload drift (relevant to the heterogeneous tiers highlighted by^29^.

Finally, to make these distinctions auditable, Table 1 (updated) contrasts Refs. 20–29 against FRAHTOS along the requested axes (convergence, overhead, adaptability, privacy). In sum, whereas^20–22,27^ are peripheral (perception/operations without scheduling), and^23–26,28,29^ each cover one facet (RL under dynamics, hybrid optimization, MA-RL scalability, or offloading under harsh networks), FRAHTOS offers an end-to-end pipeline that unifies representation learning, clustering, federated updates, and dual-track scheduling, thereby establishing originality beyond incremental hybridization.

**Table 1:** Comparison of Algorithms, Architectures, and performance measures for task allocation techniques in fog Computing.

Ref (Year)	Problem & Method	Domain	Convergence behavior	Overhead (comp/comm)	Adaptability to heterogeneity & dynamics	Key gap vs. FRAHTOS
^24^ ** 2020**	DQN for dynamic job-shop with AGV	Manufacturing scheduling	Convergence can be fragile; improved vs. heuristics	Training cost; no comm model	Adapts to queues/travel times; limited scalability	Centralized RL, no federation/representation learning; no fog heterogeneity
^22^ ** 2023**	Multimodal DL for pilot situation awareness	Human factors/ITS	Supervised; stable	High compute	Not about scheduling	No allocation/offloading, no RL/FL
^25^ ** 2023**	Hybrid knowledge + data lot-streaming scheduling	Hybrid flowshop	Optimization converges; heuristic learning aided	Compute overhead moderate	Handles dynamic orders; limited cross-site heterogeneity	No FRL/PSO–GA integration; no privacy/federation; not IoT–Fog
^21^ ** 2024**	Image transformation for defect ID (DL)	High-voltage diagnostics	Supervised; stable	Moderate–High compute	Not about fog/IoT scheduling	Domain-specific vision; no resource/task allocation
^23^ ** 2024**	RL to intervene in negative emotion contagion	Social networks	Policy-learning under non-stationarity	Moderate compute	Shows RL coping with dynamic networks	Different domain; no clustering, no federated RL, no edge constraints
^29^ ** 2024**	Task offloading & resource allocation in satellite–terrestrial networks	IoT/Edge offloading	Iterative schemes; stable under constraints	Comm-aware; link-limited	Designed for strong heterogeneity across tiers	No federated RL + clustering; different architecture than fog tri-tier
^20^ ** 2025**	Joint scene-flow + moving-object segmentation (deep multi-task)	ITS perception (LiDAR)	Supervised; stable if data rich	High compute; no comm model	Not about scheduling/offloading	Perception-focused; no task scheduling, no FRL/PSO–GA/FL
^26^ ** 2025**	Multi-objective fuzzy optimization for flight scheduling	Transportation ops	Solver-level convergence	Solver complexity; no comm	Balances multi-objectives; limited to static models	No learning/federation/clustering; not edge-constrained
^27^ ** 2025**	Economic optimization of integrated energy systems	Energy systems	Optimization convergence	Model/solver overhead	System-level adaptability (energy), not tasks	Not task/offloading; no RL/FL; peripheral to fog
^28^ ** 2025**	Scalable multi-agent RL for routing	Networking (routing)	Addresses MA-RL convergence & scalability	High compute; distributed; comm among agents	Strong for dynamic, large-scale graphs	Routing (network layer) not compute scheduling; no VAE/GMM compression

## The structure model and formulation of the problem

**Fig. 3:**
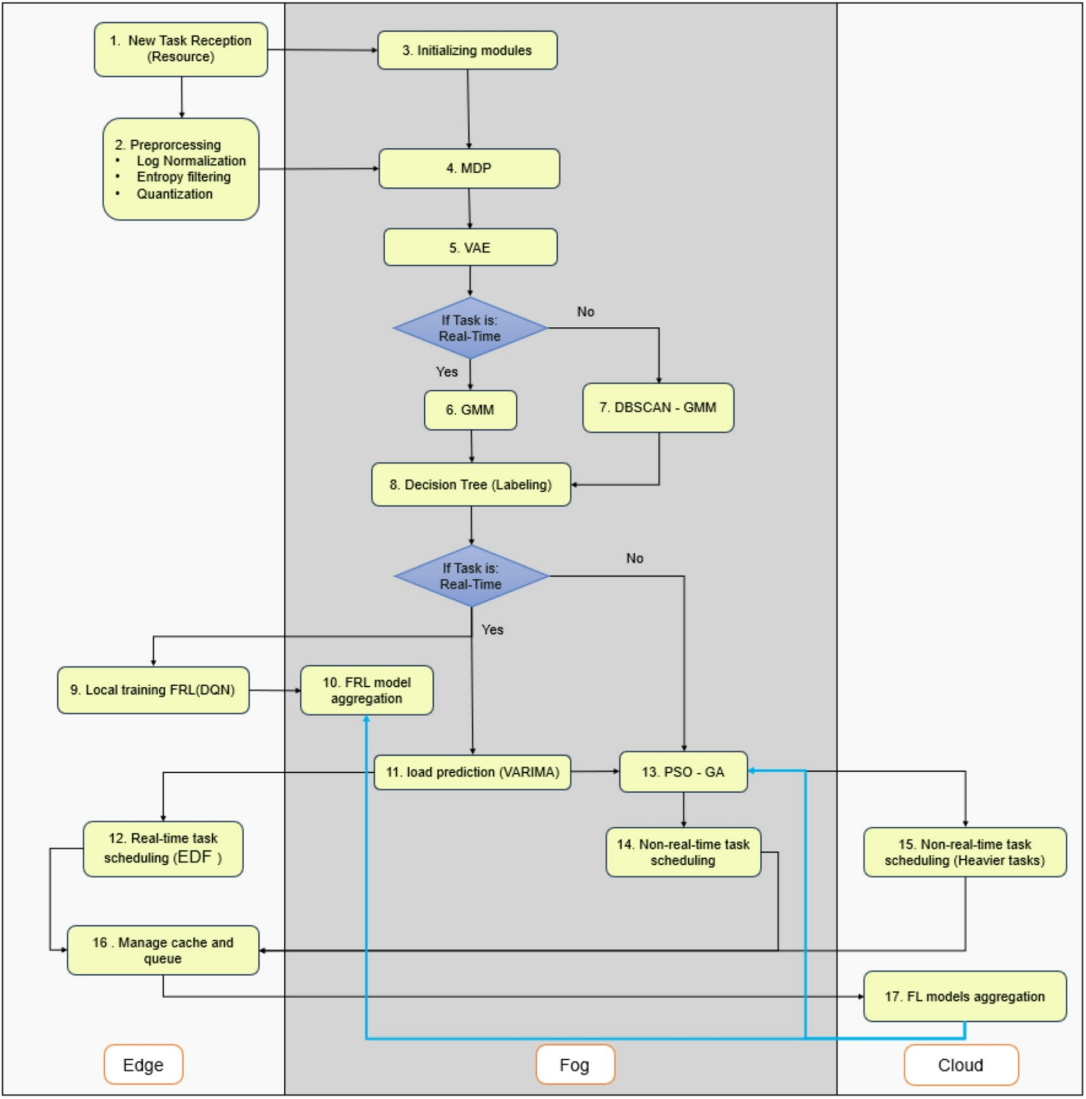
A summary of the FRAHTOS framework’s procedures for scheduling tasks in fog computing, commencing with task preprocessing and clustering, progressing to real-time (FRL-based) and non-real-time (PSO-GA) scheduling, and culminating in the integration of EDF scheduling with VARIMA load prediction.

This paper introduces an adaptive and integrated framework for task allocation in fog computing environments, aimed at concurrently optimizing multiple objectives: minimizing latency, enhancing energy efficiency, increasing allocation success rates, and ensuring scalability in extensive networks. The suggested methodology originates with multi-objective decision modeling and addresses resource heterogeneity through data preparation and distributed clustering. Subsequently, work allocation both real-time and non-real-time is executed in a customized manner utilizing federated and hybrid algorithms, while task flow management, alongside resource scheduling and forecasting, guarantees stability and optimal system performance. Figure 3 summarizes the steps of the proposed FRAHTOS technique.

### Modeling the problem

The proposed framework analyzes task allocation in dynamic and heterogeneous fog computing environments as a MDP for optimal management. The state space in this model comprises a combination of task attributes ($\:{T}_{f}$) and the resource characteristics of nodes ($\:{N}_{f}$). The task attributes comprise computational complexity, time delay, arrival rate, energy consumption, and allocation success rate, whereas the node attributes consist of processor capacity, memory, and bandwidth^30^. This combination offers a precise representation of the system state ($\:{S}_{t}$) at any moment and underpins allocation of tasks decisions.

1 $$\:{S}_{t}=\left\{{T}_{f},{\:N}_{f}\:\right\}=\left\{{D}_{t},\:{L}_{t},\:{C}_{t},{R}_{t},{E}_{t},\:{I}_{t},\:{P}_{t},\:{\mu\:}_{CPU},\:{\mu\:}_{RAM},\:{\mu\:}_{BW}\right\}$$

The action space comprises three essential components: (1) task allocation to a designated node ($\:{a}_{alloc}$), (2) scheduling of task execution based on priority ($\:{a}_{sch}$), and (3) resource management to modulate load and optimally distribute processing resources ($\:{a}_{res}$). Collectively, these measures facilitate adaptability to environmental fluctuations and sustain system efficacy^31^.

2 $$\:{A}_{t}=\left\{\left({a}_{alloc},{a}_{sch},{a}_{res}\right)\right|\:{a}_{alloc}\in\:\:{A}_{alloc}\:,\:{a}_{sch}\in\:\:{A}_{sch}\:,{a}_{res}\in\:\:{A}_{res}\}$$

The reward function in the MDP model is structured to simultaneously pursue numerous optimization targets, including decreasing latency, reducing energy consumption, enhancing allocation success rate, and increasing resource efficiency. In real-time operations, latency is the primary criterion, whereas in non-real-time tasks, minimizing energy consumption and optimizing resource use are paramount^32^. The weighting of the reward function elements is adaptively modified to align with the priorities of each task type.

3 $$\:{R}_{t}\left({s}_{t},\:{\:a}_{t}\right)=\:{w}_{1}.\:{r}_{lat}\left(\:{L}_{t},{D}_{t}\right)+\:{w}_{2}.\:{r}_{e}\left({E}_{t}\right)+{w}_{3}.\:{r}_{p}\left({P}_{t}\right)+\:{w}_{4}.\:{r}_{res}\left(\:{\mu\:}_{CPU},\:{\mu\:}_{RAM},\:{\mu\:}_{BW},{a}_{res}\right)$$

The function incorporates four elements to accomplish multi-objective optimization. The latency reward $\:{r}_{lat}\left(\:{L}_{t},{D}_{t}\right)$ promotes allocations in which the task delay $\:{L}_{t}$ is less than the deadline $\:{D}_{t}$, increasing as $\:{L}_{t}$ diminishes and imposing penalties for deadline breaches to guarantee ultra-low latency for time-sensitive applications. The energy reward $\:{r}_{e}\left({E}_{t}\right)$ enhances efficiency by incentivizing reduced energy consumption $\:{E}_{t}$ in relation to the maximum permissible energy $\:{E}_{max}$, hence assisting energy-constrained nodes. Automatic design of generative heuristics for distributed flowshop scheduling, demonstrating the advantage of generative/learning methods^33^.

The proposed framework establishes a direct connection between system observations and allocation decisions via the interaction of its three fundamental components within the MDP. The state space is characterized by a synthesis of task-level properties (such as computational complexity, arrival rate, deadline, and energy consumption) and node-level resources (including CPU capacity, memory, and bandwidth), thus encompassing both workload requirements and system heterogeneity. The action space encompasses three choice types: (i) allocating a task to a designated node, (ii) determining a scheduling sequence based on priority or deadline, and (iii) modifying resource utilization to equilibrate load distribution among nodes. The reward function synthesizes these components by incentivizing allocations that attain low latency, high task completion, and energy efficiency, while imposing penalties for deadline breaches or excessive resource use.

### Preprocessing

The proposed framework develops a multi-stage preprocessing module to prepare input data for task assignment in heterogeneous fog computing environments. This section aims to standardize, compress, and simplify the data to enhance the efficiency of subsequent processes, including clustering, assignment, and reinforcement learning. The preprocessing module aims to maintain essential information, minimize computational and communication burdens, and enhance the system’s adaptability to environmental variations.

- Initially, the input data from the state space $\:{S}_{t}$, encompassing task features and node resources, undergo *logarithmic normalization* to standardize the varying feature sizes. This approach compresses substantial values and amplifies minor data, hence rendering clustering and learning more stable^34^. The formula for logarithmic normalization is defined as follows.
  4 $$x_i^{\prime}=log\bigg(1+\frac{x_i-x_{min}}{x_{max}-{x_{min}}+10^{-6}}\bigg)$$

$\:{x}_{i}^{{\prime\:}}$ represents the normalized value, $\:{x}_{i}$ denotes the raw value, while $\:{x}_{min}$ and $\:{x}_{max}$ signify the minimum and maximum feature values inside the dataset, respectively. Annual multi-objective optimization model for chain seal scheduling that explores the trade-off between objectives (efficiency/risk/resources)^35^. This equation eliminates negative values by adding 1 into the normalization fraction, so converting the data into a positive, compact range of [0, 1], which is appropriate for processing in fog computing.

- *Entropy-based compression* is employed to minimize redundancy and eradicate useless features. During this procedure, traits characterized by low entropy and minimal contribution to decision-making are discarded^36^. The entropy of each feature $\:{x}_{i}$ is computed using following equation.

5 $$\:H\left({x}_{i}\right)=-\sum_{j=0}^{k}p\left({x}_{ij}\right).{\text{log}}_{2}\left(p\right({x}_{ij}\left)\right)$$

Thresholding and feature elimination based on entropy are crucial steps in this process to diminish data dimensionality. The feature removal criterion $\:{x}_{i}$ is applied by comparing the entropy of each feature $\:H\left({x}_{i}\right)$ with the threshold $\:{H}_{th}$, if the entropy is below the threshold ($\:H\left({x}_{i}\right)<\:{H}_{th}$), the feature is eliminated. Subsequently, *adaptive quantization* is executed to diminish computational complexity and transmission overhead. This technique transforms continuous features into discrete values and is calibrated based on the data’s variance^37^. Levels are assigned and the quantization step is calculated as follows.

6 $$\:{\varDelta\:}_{i,j}=\:\frac{{x}_{i,max}-{x}_{i,\:\:min}}{k}\:\cdot\:\frac{1}{p\left({x}_{ij}\right)}$$

The variable steps $\:{\varDelta\:}_{i,j}$ are computed according to data density, partitioning the feature domain into k intervals, with k assumed to be 16, and $\:p\left({x}_{ij}\right)$ denotes the probability of data inside interval j in relation to the aggregate number of samples^38^.

The quantity of Gaussian components is established by integrating statistical model selection with system-level analysis. GMM with K∈ {4,8,12,16,20,24} were trained using VAE latent representations, and the Bayesian Information Criterion (BIC) was assessed using the mean silhouette coefficient. Both metrics indicated a critical threshold at K = 16: the BIC attained its minimum, while the silhouette score plateaued at elevated values of K, suggesting diminishing efficacy and possible over-segmentation. In addition to statistics, K = 16 signifies the operational interpretation of environment, which consists of 2 task types (real-time/non-real-time) multiplied by 4 levels of workload difficulty (low, medium, heavy, and very heavy). Two source rows (edge/fog) yield sixteen interpretable prototypes that the scheduler may consistently associate with the EDF priorities and PSO-GA parameters. FRAHTOS sustains stability for K within the range of 12 to 20 and exhibits analogous performance patterns; therefore, K = 16 was incorporated into Eq. (6) for the sake of repeatability.

### Distributed clustering and self-supervised labeling

The proposed approach employs a feature compression process and distributed clustering to diminish data dimensionality and enhance decision-making in intricate and diverse fog computing environments. This module, following initial preprocessing, reduces computational complexity in allocation and communication overhead by diminishing data dimensionality. This section’s structure is formulated to address the complexity and heterogeneity of the environment by segregating tasks through the clustering of the GMM for real-time tasks, integrating it with the DBSCAN clustering for non-real-time tasks, and subsequently executing labeling tasks by enhancing the decision tree.

In the proposed architecture, clustering is essential for minimizing the search space and facilitating effective task classification. VAE are utilized for feature compression, converting high-dimensional IoT task data into concise latent vectors that preserve critical attributes while reducing communication and processing expenses. The latent vectors are next analyzed using GMM, which execute probabilistic clustering and are particularly effective in dynamic contexts due to their capacity for soft task allocations across clusters. DBSCAN is utilized for non-real-time tasks to detect dense task clusters without necessitating a predetermined number of clusters, which is beneficial in extensive and heterogeneous networks. Nonetheless, these methodologies have trade-offs: VAEs necessitate substantial training effort and may incur reconstruction errors, GMM presupposes data distributions that may not accurately reflect actual IoT trends, and DBSCAN can be sensitive to parameter configurations, such as neighborhood radius. Notwithstanding these constraints, the synergistic application of VAE/GMM and DBSCAN provides an effective equilibrium between scalability, flexibility, and clustering precision in extensive IoT systems, rendering them appropriate foundational components for FRAHTOS.

#### Using VAE for feature compression

The suggested approach identifies the management of high-dimensional data and diverse structures as a major difficulty in task allocation. Scalable multi-agent RL can efficiently solve large-scale routing problems and address convergence/scalability challenges^39^. Offloading and resource allocation in integrated satellite-terrestrial networks, adapting to heterogeneity and link constraints^40^. To address this issue, the Adaptive Variable Autoencoder (ADAPTIVE VAE) is employed to transform preprocessed feature vectors into low-dimensional latent vectors. This compression diminishes transmission overhead and enhances computational performance in resource-limited nodes.

Adaptive VAE is utilized subsequent to non-destructive preprocessing (normalization/winsorization) that preserves task-related semantics. Although every autoencoder-based compression is inherently lossy, proposed method specifically regulates the rate-distortion trade-off to maintain planning-related information. (7) Specifically, the evidence lower bound (ELBO) optimizes a ($\beta$) variational autoencoder (VAE).

7 $$\:{\mathcal{L}}_{ELBO}=\:{E}_{{q}_{\varphi\:}\left(z|x\right)}\left[\text{log}{p}_{\theta\:}\left(z\mid x\right)\right]-\beta\:{D}_{KL}\left[{q}_{\varphi\:}\left(z\mid x\right)\parallel p\left(z\right)\right]$$

where $\beta$ and the hidden dimension $\:{d}_{z}$ adjusted via validation to attain a specified fidelity. (2) implementing early halting based on reconstruction loss to prevent over compression; and (3) conducting two further fidelity assessments beyond reconstruction:

- Feature-level fidelity: the normalized mean squared error (MSE) and cosine similarity between the original x features and the reconstructions $\:\widehat{x}$ are assessed.
- Task-level fidelity: downstream clustering assignments (GMM/DBSCAN) with and without VAE are compared using normalized mutual information (NMI), adjusted Rand index (ARI), and silhouette score. If fidelity diminishes beyond a minor threshold, $\beta$ or $\:{d}_{z}$ escalates.

Elimination studies indicate that employing adaptive VAE does not compromise scheduling outcomes: clustering purity and assignment stability remain consistent, while communication and training overheads are diminished due to dimensionality reduction.

The VAE architecture comprises an encoder featuring three layers with ReLU activation and a decoder structured inversely. The size and quantity of neurons in each layer are established according to the input collected from the preprocessing phase. Consequently, based on the richness of the input data and the resource availability of the node, the VAE architecture is adaptive and operates in one of three modes, using 6, 7, or 9 dimensions as output dimensions, contingent upon the features derived from preprocessing. Consequently, the dimensions of the layers will alter^40^. The encoder design in these instances comprises intermediate layers of (112,56) or (96,48) neurons, respectively. The ReLU activation function in intermediate layers models nonlinear relationships, making it appropriate for heterogeneous data. In all three instances, the encoder’s output latent space will be four-dimensional.

8 $$h_1(x)=RELU(W_1x+b_1)$$

9 $$h_2{x}=RELU(W_2 h_1+b_2)$$

10 $$log\:\sigma_\phi^2(x)=W_\sigma h_2+b_\sigma\mu_\phi(x)=W_\mu h_2+b_\mu$$

Upon traversing the encoder layers, two distinct vectors, the mean $\:{\mu\:}_{\varphi\:}\left(x\right)\:$ and the logarithm of the variance $\:\text{log}{\sigma\:}_{\varphi\:}^{2}\left(\text{x}\right)$, are derived for the parameters of the latent Gaussian distribution in the space $\:{R}^{4}$. Subsequently, the reparameterization technique is employed to conduct latent sampling, resulting in the latent vector z, which serves as a compressed representation of the integrated state of the node and the task, while maintaining the differentiability of the random process.

11 $$\:z={\mu\:}_{\phi\:}\left(x\right)+{\sigma\:}_{\phi\:}\left(x\right)\cdot\:\eta\:\:\:\:\:\:\:\:\:\:\:\:\:\:\eta\:\sim N\left(0,I\right)\:\:,\:\:\:{\sigma\:}_{\phi\:}\left(x\right)=exp\left(\frac{1}{2}{log}{\sigma\:}_{\phi\:}^{2}\left(x\right)\right)$$

where $\eta$ represents a random noise characterized by a standard normal distribution with a mean of 0 and a covariance matrix of I. The decoder architecture is the inverse of the encoder and seeks to reconstruct the latent vector z into the input space to maintain essential information for clustering and task allocation^41^. The decoder output comprises 6, 7, or 9 linear neurons, accordingly, which generate an estimate $\:\widehat{x}$ of the input characteristics x.

12 $$\:{h}_{4}\left(z\right)=ReLU\left({W}_{4}{\cdot\:h}_{3}\right(z)+{b}_{4})$$

13 $$\:\widehat{x}={W}_{5}{h}_{4}+{b}_{5}$$

The loss function and optimization in Adaptive VAE $\:{\mathcal{L}}_{VAE}$ are crucial for learning process the model to compress data and produce a 4D latent space. The framework comprises two components: the reconstruction loss $\:{\mathcal{L}}_{recon}$, employing a mean squared error function for continuous data, and the KL-Laban divergence loss $\:{\mathcal{L}}_{KL}$, which aligns the latent distribution $\:{q}_{\varphi\:}\left(z\right|x)$ with the standard distribution N (0, I) and represents the data’s heterogeneity, as determined by the subsequent Eq. 

14 $$\:{\mathcal{L}}_{VAE}=\:{\mathcal{L}}_{recon}+\:\:\beta\:\cdot\:{\mathcal{L}}_{KL}=\:\frac{1}{2}\sum_{i=1}^{{D}_{input}}{\left|x-\widehat{x}\right|}^{2}+\frac{1}{2}\sum_{j=1}^{4}({{\mu\:}_{\phi\:}\left(x\right)}^{2}+{{\sigma\:}_{\phi\:}\left(x\right)}^{2}-{log}{\sigma\:}_{\phi\:}^{2}\left(x\right)-1)$$

$\:{D}_{input}$ represents the input dimension, whereas β is a coefficient that enhances the optimization and compression accuracy of the VAE by adjusting it with other parameters, including the learning rate within the interval [0.001, 0.005] and the number of neurons. For non-real-time tasks, $\beta$ is set at 1.5, whereas for real-time tasks, it adjusts according to the arrival rate as $\:\beta\:=0.1\cdot\:min(\frac{{I}_{t}}{10},1)$. VAE training is conducted federatively across nodes. Each node calculates the Adam stochastic gradient descent, $\:{\nabla\:}_{\varphi\:\:}{\mathcal{L}}_{VAE}$, representing the gradient of the loss function $\:{\mathcal{L}}_{VAE}$ concerning the encoder parameters, and $\:{\nabla\:}_{\theta\:\:}{\mathcal{L}}_{VAE}$, denoting the gradient of the loss function $\:{\mathcal{L}}_{VAE}$ regarding the decoder parameters, utilizing local data such as task and node features, while synchronizing with the Top-K clustering algorithm and selecting 10% of the largest gradients. This streamlined architecture minimizes communication overhead and guarantees scalability. It is appropriate for low-memory nodes and diminishes the computation from a 9-dimensional space to a 4-dimensional one.

**Figure:**
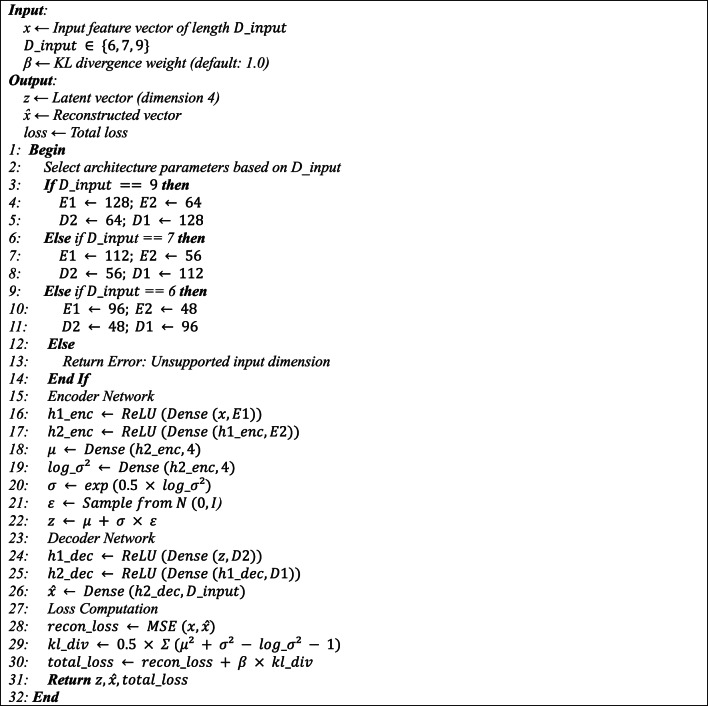
**Algorithm 1**. Adaptive Variational Autoencoder for Task Feature Compression.

#### GMM-based real-time clustering with soft assignment

The proposed framework employs GMM tailored for a task group facing stringent time restrictions and real-time task categorization, aiming to enhance assignment accuracy, minimize latency, and maintain network stability. In contrast to conventional hard assignment algorithms like K-means, GMM determine the likelihood of each task belonging to various clusters through numerous Gaussian distributions, facilitating adaptive assignment under dynamic situations.

The number of clusters k is adaptively determined according to the real-time task arrival rate denoted as $\:\lfloor5+5\cdot\:min(\frac{{I}_{t}}{10},1)\rfloor$. These vectors establish the foundation for clustering within the probability space^42^.

- The GMM training procedure relies on the Expectation-Maximization (EM) method. During the Expectation Step (E-step), the value $\:{\gamma\:}_{ik}$, representing the membership probability of vector $\:{z}_{i}$ in cluster k, is computed utilizing the multivariate normal density function as follows.

15 $$\:{\gamma\:}_{ik}=\frac{{\pi\:}_{k}\cdot\:N\left({z}_{i}|{\mu\:}_{k}\:,\:{\sum\:}_{k}\right)}{\sum_{j=1}^{k}{\pi\:}_{j}\cdot\:N\left({z}_{i}|{\mu\:}_{j}\:,\:{\sum\:}_{j}\right)}$$

$\:N\left({z}_{i}|{\mu\:}_{k}\:,\:{{\Sigma\:}}_{k}\right)$ denotes the Gaussian distribution, with parameters $\:{\mu\:}_{k}$, $\:{{\Sigma\:}}_{k}$, and $\:{\pi\:}_{k}$ representing the mean, covariance, and weight of cluster k, respectively. These parameters are initialized using K-means to enhance the speed and stability of the convergence process^43,44^. This likelihood enables the probabilistic allocation of real-time tasks to several clusters, enhancing flexibility in dynamic contexts.

- During the maximizing step (M-step), the parameters $\:{\mu\:}_{k}$, $\:{{\Sigma\:}}_{k}$, and $\:{\pi\:}_{k}$ are revised using the subsequent equations to optimize the log-likelihood of the data.
  16 $$\mu_k=\frac{\sum_{i=1}^N \gamma_{ik}z_i}{\sum_{i=1}^N \gamma_{ik}}$$
  17 $$\sum\nolimits_k=\frac{\sum_{i=1}^N \gamma_{ik}(z_i-\mu_k)(z_i-\mu_k)^T}{\sum_{i=1}^N \gamma_{ik}}$$
  18 $$\pi_k=\frac {\sum_{i=10}^N \gamma_{ik}}{N}$$

In these equations, N denotes the total number of tasks. Following the convergence of the GMM, cluster refinement is executed utilizing the soft membership probability $\:{\gamma\:}_{ik}$ to allocate tasks to suitable nodes in real time. For each task represented by vector $\:{z}_{i}$, the cluster $\:{k}^{*}$ exhibiting the highest probability $\:{\gamma\:}_{ik}$ is chosen, while non-zero probabilities for other clusters are also taken into account to facilitate an alternative assignment should the primary node assignment prove unsuccessful.

#### Using DBSCAN and GMM for non-real time clustering

This section of the proposed framework employs a hybrid clustering approach that integrates DBSCAN and the GMM to mitigate the intricacies of resource allocation and optimization for non-real-time tasks. This amalgamation offers the benefits of density-based clustering and statistical modeling to concurrently address imbalanced and nonlinear data^45^.

Initially, the DBSCAN algorithm serves as a preliminary cluster, examining the latent vectors derived from the VAE. Utilizing the parameters r (neighborhood radius) and MinPts (minimum neighboring points), DBSCAN discerns dense clusters and eliminates low-density points as noise. This component can recognize high-density structures and efficiently segregate heterogeneous data without requiring the specification of the number of clusters. The value of r is established according to the standard deviation of the data, with MinPts set to ⌈log(N)⌉ to ensure scalability. The criteria for membership and cluster formation in DBSCAN are articulated as follows.

19 $$\mid \{{z}_{j}:{\parallel{z}_{i}-{z}_{j}\parallel}_{2}\le\:r\} \mid \ge\:MinPts$$

In the second stage, the GMM model is executed on the valid clusters derived from DBSCAN to refine the clusters and enhance the accuracy of substructure separation. The process is refined using the expectation-maximum (EM) algorithm to compute the soft membership probability $\:{\gamma\:}_{ik}$ from Eq. (15) for each vector $\:{z}_{i}$, while the parameters $\:{\mu\:}_{k}$, $\:{{\Sigma\:}}_{k}$, and $\:{\pi\:}_{k}$ are adjusted in accordance with Eqs. (16), (17), and (18), respectively. The characteristics of these probabilities facilitate the adaptable allocation of non-real-time tasks, which is essential for scalability at elevated task volumes.

#### Using a decision tree to create labels

The proposed method employs a decision tree as a lightweight supervised learning technique to enhance task allocation accuracy and minimize computing overhead during the task cluster refining and labeling phase. This component enhances the preliminary unsupervised clustering procedure (GMM) and is essential for associating the compressed latent vectors $\:{z}_{i}$ with the cluster labels $\:{C}_{k}$.

The decision tree structure is constructed from the latent vectors produced by the VAE and the initial GMM clustering results, utilizing the CART (Classification and Regression Tree) technique. The CART method in the proposed system employs training data $$\:\begin{array}{c}N\\\:i=1\end{array}\left\{\left({z}_{i},{C}_{k}\right)\right\}$$, utilizing the data features $\:{z}_{i}$ to forecast the cluster label $\:{C}_{k}$, with the partitioning criterion being the minimization of the Gini index, defined as follows.

20 $$\:Gini\left(m\right)=1-\sum_{k=1}^{k}{p}_{mk}^{2}$$

where $\:{p}_{mk}$ denotes the likelihood that the samples from node m are associated with cluster $\:{C}_{k}$. At each node, the feature $\:{z}_{ij}$ and the threshold τ must be selected to minimize the weighted sum of the Gini coefficients in the two subnodes, hence enhancing the accuracy of the classification. This recursive division persists until a stopping requirement is met, such as a depth of 5 for real-time or 8 for non-real-time, or a minimum sample size within the node.

21 $$\:\left({j}^{\text{*}},{\tau\:}^{\text{*}}\right)=arg\underset{j,\tau\:}{{min}}\bigg(\frac{\left|{m}_{left}\right|}{\left|m\right|}Gini\left({m}_{left}\right)+\frac{\left|{m}_{right}\right|}{\left|m\right|}Gini\left({m}_{right}\right)\bigg)$$

In the aforementioned equation, $\:{m}_{left}$ and $\:{m}_{right}$ denote the points in the left and right subnodes, respectively, whereas the ideally selected feature $\:{j}^{*}$ corresponds to the feature with the optimal threshold $\:{\tau\:}^{*}$. Subsequent to the initial tree construction, CART does tree pruning utilizing the cost-complexity criterion $\:{R}_{\alpha\:}\left(T\right)$ to mitigate tree overgrowth and manage computational demands, particularly in real-time nodes with constrained memory.

22 $$\:{R}_{\alpha\:}\left(T\right)=\sum_{m\:\in\:T}Gini\left(m\right)\:+\:\alpha\:\left|T\right|$$

where $\mid T \mid$ denotes the quantity of leaf nodes and α regulates the complexity weight. This pruning eliminates superfluous nodes, and the definitive label for each vector $\:{z}_{i}$ is established by navigating the decision path from the root to the leaf node. At every non-leaf node, the conditions $\:{m}_{left}$ and $\:{m}_{right}$ are evaluated until the leaf node $\:{m}_{leaf}$ is attained, and the label $\:{C}_{k}$ is determined by the probability that the majority of samples correspond to which leaf nodes $\:{m}_{leaf}$. Upon completion of training, the decision tree is employed to assign labels to new tasks with input $\:{z}_{i}$, predicting the cluster label $\:{C}_{k}$ by navigating the decision route. This approach is expeditious for real-time activities, as the shallow tree minimizes computational demands. For non-real-time tasks, labeling is conducted on central nodes with elevated$\:{\mu\:}_{CPU}$ to guarantee scalability for data-intensive operations.

**Figure:**
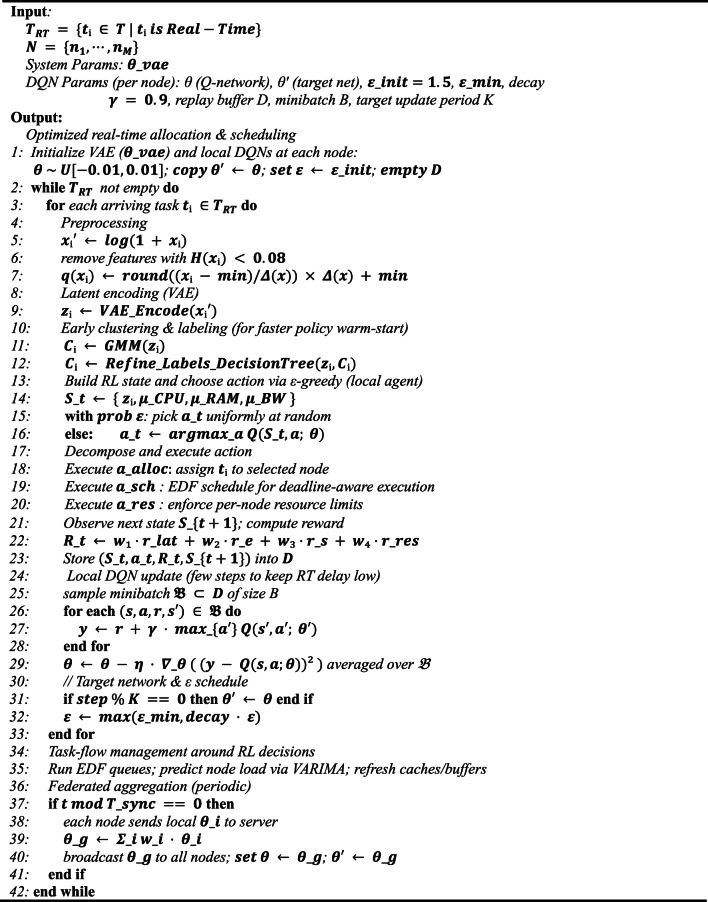
**Algorithm 2** Task scheduling in real-time for fog computing using the FRAHTOS framework

### Real-time task allocation using FRL

The suggested architecture conceptualizes real-time task allocation with stringent time restrictions as a sequential choice issue within the FRL framework, represented as a Markov choice Process (MDP). A lightweight model utilizing Deep Distributed Q-Network (DQN) is employed to accommodate diverse nodes and dynamic surroundings. This model is structured to facilitate decentralized task allocation policy learning at each node, with the global model being updated by federated aggregation; hence, communication overhead is minimized and the necessity for raw data interchange is obviated. The task scheduling procedure for real-time workloads is executed utilizing the proposed FRAHTOS framework as delineated in Algorithm 2.

This model represents decision-making as a series of states, actions, and rewards, facilitating the learning of the optimal allocation policy in response to environmental changes. For each local agent in the fog nodes, the state space is modified by integrating the compressed latent vector derived from the variable autoencoder (VAE) with the node’s computational resource state in the state space as $\:{S}_{t}=\left\{{z}_{t},\:{\mu\:}_{CPU},\:{\mu\:}_{RAM},\:{\mu\:}_{BW}\right\}$. This section defines the action space and reward function in a manner analogous to the reward function of the overall MDP inside the proposed architecture, aiming to achieve numerous optimization targets concurrently. This function employs Eq. (3) to compute the reward of the DQN Network.

During the training and allocation process in local nodes, a shallow Q network is utilized, comprising three layers with neuron counts of 8, 16, and 4, respectively (4→16→8). To satisfy the fundamental computational prerequisites. This network predicts the value of the action-value function $\:Q(s,a;\theta\:)$ according to the Eq. (23). To expedite convergence in real-time tasks, local nodes employ early clustering to establish policies. The Q function serves as a preliminary guide, with its network parameters θ randomly initialized from a uniform distribution inside the interval [−0.01, 0.01].

23 $$\:Q(s,a;\theta\:)\approx\:\mathbb{E}\left[R\right(s,a\left)\right]$$

It executes action selection utilizing the ε-grid policy as delineated by the Eq. (24). The value of ε is first established as 1.5 to facilitate investigation and progressively diminishes to a lesser value.

24 $$\:\pi\:\left({s}_{t}\right)=\left\{\begin{array}{c}\begin{array}{cc}Random\:&\:\:\:\:\:\:\:\:\:\:\:\:\:\:\:\:\:\:\:\:\:\:\:\:\:\:\:\:\:p=\epsilon\:=0.05\end{array}\\\:\begin{array}{cc}arg\underset{a}{{max}}Q({s}_{t},a;\theta\:)\:\:\:\:\:\:\:\:\:&\:\:\:\:\:p=1-\epsilon\:\end{array}\end{array}\right.$$

The action-value function is updated with the Bellman update relation as follows.

25 $$\:Q\left({s}_{t},{a}_{t};\theta\:\right)\approx\:\mathbb{E}\left[{R}_{t}+\gamma\:arg\underset{{a}_{t+1}}{{max}}Q\left({s}_{t+1},{a}_{t+1};{\theta\:}^{{\prime\:}}\right)\right]$$

where $\gamma$ represents the discount factor and $\:{\theta\:}^{{\prime\:}}$denotes the characteristics of the target network. The value of $\gamma$ is established at 0.9. This value establishes an appropriate equilibrium between focus on immediate and future rewards, hence enhancing training stability in turbulent contexts. Local nodes compute the reward according to the local characteristics of Eq. (3), utilizing prior allocation feedback to refine the policy. The policy update at the local nodes is executed by training the network, utilizing the stored experiences $\:\left({s}_{t},{a}_{t},{R}_{t},{s}_{t+1}\right)$ in the experience buffer through the DQN loss function as follows.

26 $$\:{\mathcal{L}}_{DQN}\left(\theta\:\right)=\mathbb{E}\left[{\left({R}_{t}+\gamma\:arg\underset{{a}_{t+1}}{{max}}Q\left({s}_{t+1},{a}_{t+1};{\theta\:}^{{\prime\:}}\right)-Q\left({s}_{t},{a}_{t};\theta\:\right)\right)}^{2}\right]$$

In real-time operations, the number of repetitions is constrained to provide minimal delay. The target network $\:{\theta\:}^{{\prime\:}}$ is adjusted periodically to ensure training stability. To achieve convergence to the global policy, local polynomial models transmit their $\:{\theta\:}_{i}$ to the central server, where federated aggregation is conducted in a weighted way.

27 $$\:{\theta\:}_{g}=\sum_{i=1}^{M}{w}_{i}{\theta\:}_{i}$$

where M represents the total number of nodes and $\:{w}_{i}$ signifies the weight of node i, determined by the ratio of its local instances. Subsequent to aggregation, the global model $\:{\theta\:}_{g}$ is disseminated to all nodes to revise the real-time task allocations and determine the optimal action $\:{a}^{*}$ is ascertained by the maximum value of the action-value function. Feedback from the assignments is utilized to modify the reward function and facilitate retraining.

### Non-real-time task allocation using PSO-GA

The proposed paradigm defines non-real-time task allocation as a multi-criteria optimization problem that concurrently optimizes energy consumption, resource efficiency, and success rate. A hybrid optimization strategy integrating PSO and GA is suggested, taking into account the distinct traits of non-real-time tasks that exhibit lower sensitivity to delays but produce greater processing demands and data volume. This framework, while guaranteeing precision and rapid convergence, leverages the capabilities of random and adaptive search to attain optimal resource allocation in fog and cloud settings. The task scheduling procedure for non-real-time workloads is executed utilizing the proposed FRAHTOS framework as delineated in Algorithm 3.

The selection of a PSO-GA hybrid optimization is driven by the synergistic advantages of the two metaheuristic methods. PSO is recognized for its rapid convergence and simplicity; nonetheless, it frequently experiences premature convergence and stagnation at local optima. Conversely, GA offer robust exploration capabilities via crossover and mutation operators; however, they generally necessitate extended convergence durations. The proposed framework combines PSO and GA, utilizing the swift search and exploitation capabilities of PSO alongside the diversity-preserving features of GA, leading to enhanced convergence speed and superior solution quality. This hybrid methodology is especially appropriate for non-real-time IoT applications, characterized by high computing demands with minimal sensitivity to latency, inside a high-dimensional search space. In comparison to single-algorithm approaches or other heuristics like Simulated Annealing or Ant Colony Optimization, PSO–GA attains a superior equilibrium between exploration and exploitation, hence ensuring scalability and energy efficiency in fog computing contexts.

The hybrid PSO-GA optimization process commences with the generation of an initial population of particles, each representing a potential solution for the allocation of task to nodes. The initial particle count is fixed at *P* = 50 in this structure. Each particle $\:{X}_{p}=\{{x}_{p1},\cdots\:,{x}_{p1}\}$ is an assignment vector, with $\:{x}_{pi}$ denoting the selection of the suitable node from M nodes to do the i-th task. Employing latent vectors for disparate clusters that induce the emergence of separate clusters inside the decision space. The particles’ starting positions and velocities are randomly initialized within the assignment space.

Clustering labels derived from GMM are utilized to enhance the quality of the initial population and accelerate convergence. The labels $\:{C}_{k}$ direct the compressed vectors $\:{z}_{i}$ towards quasi-optimal clusters, so enhancing the proximity of the initial allocation of each particle to processing nodes with suitable capacity. This results in the creation of more distinct clusters within the latent space. The initial evaluation of particle quality is defined by the objective function $\:f\left({X}_{p}\right)$, which is based on the Markov reward function in Eq. (3). Establishing this goal function according to the MDP action space enables the algorithm to evaluate near-optimal assignments from the outset of the search phase.

During the PSO optimization phase, the changes to particle location and velocity are structured to ensure that the task assignment search is initially directed by information derived from the MDP action space. This approach facilitates an adaptive search strategy towards quasi-optimal assignments and enhances convergence speed. The velocity of each particle $\:{V}_{p}$ is determined by integrating the optimal local position $\:{P}_{best,p}$ and the optimal global position $\:{G}_{best}$.

28 $$\:{V}_{p}^{t+1}=w{V}_{p}^{t}+{c}_{1}{r}_{1}\left({P}_{best,p}-{X}_{p}^{t}\right)+{c}_{2}{r}_{2}({G}_{best}-{X}_{p}^{t})$$

where $\:w=0.7\:\:\:,\:{c}_{1}={c}_{2}=1.5$, and $\:{r}_{1},\:{r}_{1}\sim U(0,\:1)$, the particle position is changed accordingly.

29 $$\:{X}_{p}^{t+1}={X}_{p}^{t}+{V}_{p}^{t+1}$$

This update guarantees concurrent exploration of the search space and utilization of existing information. To accommodate the scaling to M = 1500 nodes and *N* = 10,000 tasks, the particle count is restricted to *P* = 50 and the iteration count to $\:{T}_{iter}=20$ to maintain computational complexity at O(N∙P∙$\:{T}_{iter}$). The output of the Variational Autoencoder and initial clustering segments the search space into discrete areas, facilitating the expedited navigation of particles toward ideal locations. The global optimal position $\:{G}_{best}$ is established via the greatest objective function criterion derived from the Markov reward function.

30 $$\:{G}_{best}=arg\underset{{X}_{p}}{\text{max}}f\left({X}_{p}\right)$$

GA operators (selection, combination, mutation) are utilized to enhance variety and optimize solutions. During the selection phase, particles exhibiting the greatest objective function values are chosen using the tournament method.

31 $$\:if\:f\left({X}_{p}\right)>f\left({X}_{q}\right)\:then\:{X}_{p}\:selected$$

Crossover is executed between the two parent particles with a probability of $\:\:{p}_{c}$=0.8.

32 $$\:{X}_{child}=\alpha\:{X}_{p1}+\left(1-\alpha\:\right){X}_{p2},\:\:\:\:\:\:\:\:\:\:\alpha\:\sim u\left(\text{0,1}\right)\:\:$$

This procedure preserves the variety of the tasks. Combination occurs at the center nodes to alleviate intensive computations from low-resource or low $\:{\mu\:}_{RAM\:}$ nodes. Mutation is implemented with a probability of $\:{p}_{m}=0.1$ to prevent entrapment in local optima.

33 $$x_{pi}=x_{pi}+\delta\:,\:\:\:\:\:\:\delta\:\sim\mathcal{N}\left(0,{\sigma\:}^{2}\right)$$

This technique preserves population variability and facilitates a more efficient exploration of the assignment space. In non-real-time activities, mutation significantly enhances assignment quality and elevates the success rate. The parameter σ = 0.1 enables this method to preserve population variety and facilitates the efficient exploration of the assignment space by PSO-GA. The objective function is established according to the Markov reward function. In non-real-time scenarios, $\:{w}_{1}$ is configured to favor a high success rate. Upon assessment, the local optimal position $\:{P}_{best,p}$ as following and global $\:{G}_{best}$ by Eq. (30) are revised for each particle.

34 $$\:if\:f\left({X}_{p}\right)>f\left({P}_{best,p}\right)\:then\:{P}_{best,p}={X}_{p}$$

The updating procedure occurs in non-real-time at the central nodes to provide maximum task scalability and minimal complexity. At this stage, the distributed aggregation method involves gathering and integrating the optimal local solutions $\:{G}_{best,i}$ from various nodes. The central server subsequently identifies the optimal global solution utilizing the goal function as outlined.

35 $$\:{G}_{glob}=arg\underset{{G}_{{best},i}}{{max}}f\left({G}_{best,i}\right)$$

Subsequent to aggregation, $\:{G}_{glob}$ is disseminated to the local nodes for the purpose of updating their allocations. Feedback from the assignments is utilized to modify the Markov-compatible objective function and enhance the solutions. This approach facilitates the concurrent optimization of numerous objectives, with a success rate high while minimizing energy consumption. The centralization of computing at primary nodes, coupled with restricted data movement, mitigates communication overhead and sustains overall system efficiency. This distributed design guarantees scalability for substantial volumes of real-time tasks.

### Management of task flow and final scheduling

A multi-stage mechanism is developed in the Task Flow Management and Final Scheduling section to enhance the allocation of real-time and non-real-time tasks in heterogeneous and scalable fog computing environments. This section integrates traditional scheduling and load forecasting techniques to improve task allocation across many performance measures, including latency, energy usage, and success rate.

Initially, work prioritizing is conducted according to the deadline. The Earliest Deadline First (EDF) method arranges tasks in ascending order according to their deadlines, $\:{d}_{i}$. This procedure operates with a time complexity of O (N log N) for N tasks. Tasks with shorter deadlines will be prioritized in allocation, particularly in real-time task distribution. To alleviate the computational burden on resource-limited edge nodes, the preprocessed features $\:{x}_{i}^{{\prime\:}}$ and cluster labels $\:{C}_{k}$ obtained from GMM facilitate the identification of analogous tasks, thereby enhancing prioritization accuracy.

The VARIMA (Vector Autoregressive Integrated Moving Average) model is employed to forecast node load and prevent overload. The time series of the load for each node, encompassing processing resources and memory, is represented as a vector $\:{y}_{t}$.

36 $$\:{y}_{t}=\sum_{i=1}^{p}{\Phi\:}_{i}{y}_{t-i}+\sum_{j=1}^{q}{\varTheta\:}_{i}{\epsilon}_{t-i}+{\epsilon}_{t}\:\:\:\:\:\:\:\:\:\:,\:\:{\epsilon}_{t}\sim\mathcal{N}(0,\sum\:)$$

A dynamic cache is employed to expedite the assignment process by selecting suitable nodes according on EDF priorities and VARIMA forecasts. The dynamic cache retains prior assignments, including the designated node $\:{g}_{i}$, in the format of a set $\:Cache\left(i\right)=\left\{\left({x}_{i}^{{\prime\:}},{C}_{k},{g}_{i}\right)\right\}$. If a new task possesses same features $\:{x}_{i}^{{\prime\:}}$ and cluster label $\:{C}_{k}$ as a previously cached task, the assignment is directed to the same node as before.

**Figure:**
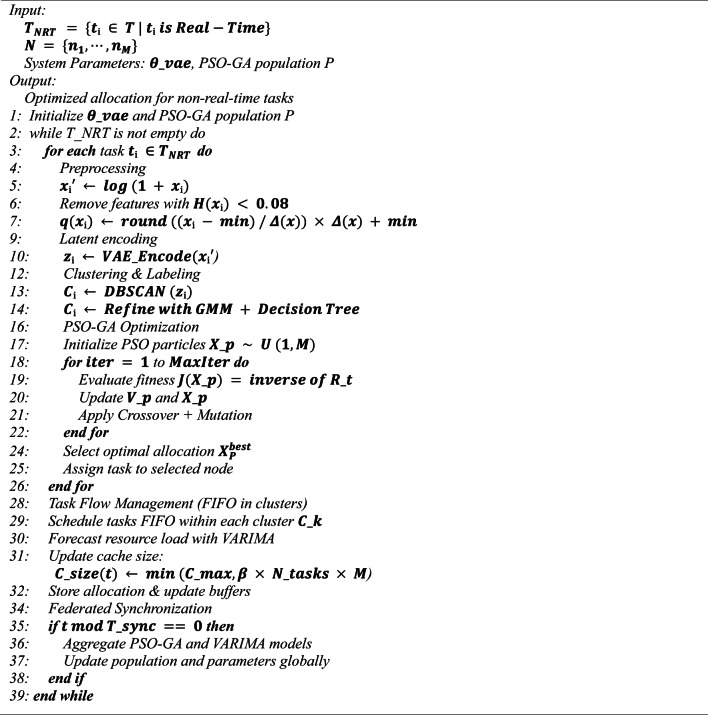
**Algorithm 3**. Task scheduling in non-real-time for fog computing using the FRAHTOS framework

This approach decreases allocation latency for real-time tasks to under 5 milliseconds and reduces computing overhead. For non-real-time processes, the cache is refreshed on the central nodes to provide scalability. A federated aggregation procedure is designed for coordinated load control and prioritization on a large scale. EDF priorities and VARIMA forecasts are aggregated from all nodes.

37 $$\:Global\:Priority=\bigcup_{i=1}^{M}{Priority}_{i}$$

38 $$\:Global\:Forecast=\bigcup_{i=1}^{M}{y}_{t,i}$$

Subsequent to aggregation, the global priorities and forecasts are disseminated to local nodes for the purpose of updating allocations. Based on global priority and global forecast, allocate $\:{g}_{i}$ to task i. This method optimizes the assignment of new tasks based on anticipated workload and scheduling precedence. The feedback from the allocations is utilized to perpetually update the EDF queues and VARIMA models.

## Experiments and results

### Evaluation criteria

This section presents the evaluation criteria employed to compare the performance of the proposed approach against other algorithms in the domains of federated learning and IoT. Each criterion is delineated individually, and its significance in assessing the efficiency and effectiveness of distributed systems is examined.

- *The average utility* metric quantifies the overall value or efficiency of an algorithm in executing IoT tasks, typically defined as a synthesis of quality of service (e.g., model accuracy) and resource efficiency (e.g., response time and energy consumption.
- *Average resource utilization* assesses the efficiency of computing resources, including CPU, memory, and network bandwidth, utilized by computing nodes within a distributed system. This metric is essential in IoT, as nodes (such as fog or edge devices) often possess limited resources, and their optimal utilization can enhance overall system performance.
- *Average task completion* quantifies the proportion of tasks successfully executed within a specified timeframe in an IoT system. This metric reflects the reliability and efficacy of an algorithm in managing computational tasks, including sensor data processing or learning model updates.
- *The estimated response latency* quantifies the duration necessary to process and reply to a request or task within an IoT system, typically expressed in milliseconds. This metric holds significant relevance in time-sensitive applications, such as smart cities or healthcare, where minimal latency guarantees real-time performance.
- *Global loss convergence* quantifies the rate of decline in the global model loss function throughout the federated learning process, reflecting the model’s accuracy and stability, typically expressed as a numerical value (ranging from 0.1 to 1).
- *Energy consumption* quantifies the energy utilized by computing nodes (such as sensors or fog nodes) during task execution or model updates, generally expressed in millijoules (mJ). In the Internet of Things, where nodes are frequently powered by batteries, minimizing energy usage is essential for prolonging device longevity.

Table 2 delineates the experimental configuration employed to assess FRAHTOS, serving to enhance reproducibility. It delineates the hardware platform, the iFogSim-2.0 simulator, and a three-tier IoT-Fog-Cloud architecture, along with network parameters (bandwidth, edge-fog and fog-cloud latencies, and packet loss), workload models (Poisson arrivals, random-walk mobility, scenario ranges), and execution protocol (1-hour simulations across 10 fixed seeds). Method-specific configurations are detailed: preprocessing, Adaptive-VAE parameters ($\:{D}_{\text{i}\text{n}\text{p}\text{u}\text{t}}$, latent size, adaptive β), clustering (GMM with K = 16 plus DBSCAN/Decision-Tree labels), FRL state and reward formulation for real-time scheduling, PSO–GA hyper-parameters for non-real-time allocation, federated aggregation frequency, queueing strategies (EDF/FIFO), VARIMA forecasting, and cache dimensions. Energy is quantified using iFogSim’s power model and expressed in millijoules (mJ).

**Table 2:** Hardware specifications and simulation parameters.

Category	Specification/Setting
Hardware	Intel Xeon 3.2 GHz (8 cores), 64 GB RAM, Ubuntu 22.04 LTS; no discrete GPU
Simulation platform	iFogSim 2.0 (Java), JDK 17
Topology	Three-tier IoT → Fog → Cloud architecture
Edge nodes (CPU/RAM)	CPU: 1.2–3.0 GHz; RAM: 1–8 GB
Fog nodes (CPU/RAM)	CPU: 2.6–3.6 GHz; RAM: 8–32 GB
Cloud	Central DC, high-capacity (no CPU bottleneck assumed)
Wireless bandwidth	10–50 Mbps (uniform per scenario)
Latency (Edge↔Fog)	2–5 ms
Latency (Fog↔Cloud)	15–25 ms
Packet loss	≤ 0.5% (background)
Task arrivals	Poisson (λ scenario-dependent); burst factor ≤ 1.2
Mobility model	Random walk (bounded area); handover cost included
Scenario ranges	Task size: 1k-50k units; Nodes: 5–50; Complexity: low/medium/high
Runtime/seeds	1 h simulated time per run; 10 runs with fixed seeds {1…10}
Energy model	iFogSim power model; idle/active measured; reporting in mJ
Preprocessing	$\:\text{log}(1+x)$, entropy filter H(x)<0.08، quantization step Δ(x)
Adaptive VAE	Input dim $\:{D}_{\text{i}\text{n}\text{p}\text{u}\text{t}}\in\:\:\{\text{6,7},9\}\:$latent $\:{d}_{z}=4$; β-VAE with adaptive β; early stopping
Clustering	GMM K = 16 (BIC elbow); DBSCAN (ε, minPts tuned per scenario) + Decision Tree labels
Real-time scheduling	FRL; state $\:{S}_{t}=\:\{x{\prime\:},\:z,\:NodeStatus\}$; reward $\:{R}_{t}\left({s}_{t},\:{\:a}_{t}\right)=\:{w}_{1}.\:{r}_{lat}+\:{w}_{2}.\:{r}_{e}+{w}_{3}.\:{r}_{p}+\:{w}_{4}.\:{r}_{res}$
Non-real-time scheduling	PSO–GA hybrid for allocation
Federated settings	FedAvg; local steps = 5; sync period $\:{T}_{\text{s}\text{y}\text{n}\text{c}}=20\:s$ simulated; batch size = 64
PSO–GA hyper-params	pop = 30; max iter = 50; inertia w = 0.7; $\:{c}_{1}={c}_{2}=1.4$; crossover = 0.8; mutation = 0.1
EDF & queues	EDF preemptive for RT; FIFO within clusters for NRT
Forecasting	VARIMA (orders tuned per scenario) for load prediction
Caching	Dynamic cache: $\:{C}_{size}=\text{m}\text{i}\text{n}({C}_{max}\:,\beta\:\times\:{N}_{task}\times\:M)$

### Analyzing experiment results

This section analyzes the experimental outcomes of the proposed approach (FRAHTOS) in comparison with reference algorithms, including FRL^38^, DDPG-AMOPG^6^, DDPG^16^, PFR-OA^23^, and GA-PSO^37^, based on seven important criteria. Each indicator is analyzed through simulations across various IoT and federated learning situations, incorporating factors such as node quantity, data sensitivity, and learning rate. The results are illustrated through graphs to facilitate a thorough comparison of the algorithms. This section aims to elucidate the strengths of FRAHTOS and identify the elements influencing its performance relative to previous approaches.

- ***Experiments 1***

This section’s experiments aim to examine the average utility metric within federated learning systems for the Internet of Things (IoT) and to compare the efficacy of the proposed approach (FRAHTOS) against existing algorithms. The average utility metric serves as a measure of the system’s overall efficiency in delivering quality services, taking into account model accuracy and resource efficiency. Evaluations are conducted across three distinct scenarios: varying the number of tasks (1000, 5000, 10000, 50000), the number of nodes (5, 10, 20, 50), and the complexity of tasks (low, medium, high). These assessments are executed in the iFogSim simulated environment and in distributed architectures, such as the 3-Tier model, to analyze the influence of various scalability and complexity dimensions on algorithm performance.

**Fig. 4:**
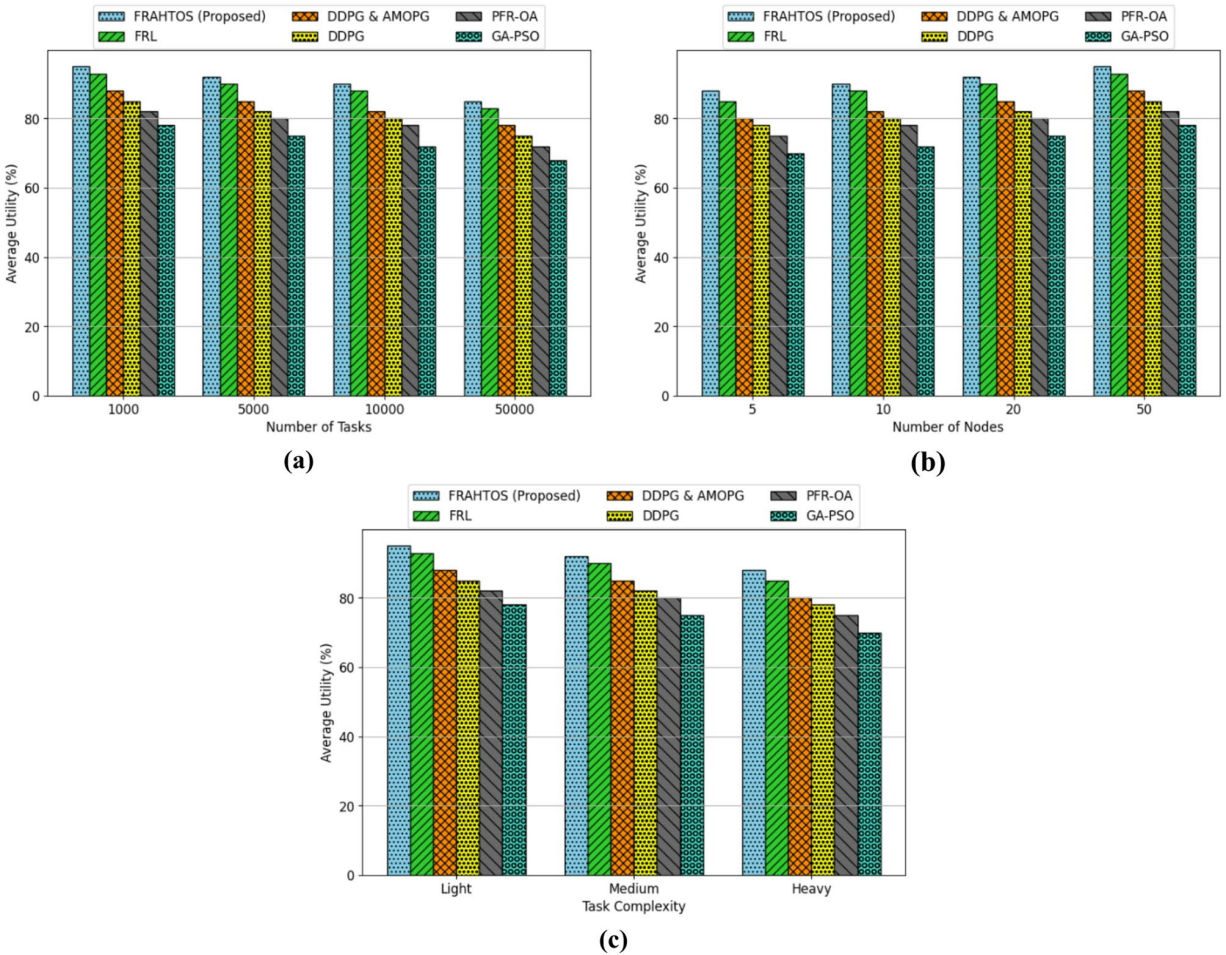
Comparison of average utility between FRAHTOS and baseline approaches over three scenarios: (**a**) differing task quantities, (**b**) differing node quantities, and (**c**) differing task complexities. FRAHTOS constantly exhibits superior utility across all scenarios.

The analysis of average utility across varying task quantities (1000, 5000, 10000, 50000), with a constant node count of 20 and tasks of moderate complexity, as depicted in Fig. 4, demonstrates that the FRAHTOS (Proposed) method consistently surpasses other reference algorithms, including FRL, DDPG & AMOPG, DDPG, PFR-OA, and GA-PSO. As the task size escalates, the utility diminishes for all algorithms, signifying scalability issues in handling substantial workloads. Specifically, FRAHTOS has sustained a utility of 95% at the 1000 task level and 85% at the 5000-task level, whereas its nearest competitor, FRL, achieves just 83% utility at the maximum task level. This advantage likely arises from characteristics such as dynamic resource allocation and astute task prioritization. The substantial decline in the efficiency of the GA-PSO algorithm (up to 68%) under a high task load suggests its unsuitability for IoT environments characterized by distributed architecture, whereas reinforcement learning-based algorithms, such as FRAHTOS and FRL, demonstrate superior capacity to manage elevated workload demands.

The second graph in Fig. 4 illustrates the impact of varying node quantities (5, 10, 20, 50) on average utility under steady-state conditions (5000 tasks of moderate complexity), indicating that an increase in node count enhances utility across all methods. Among the options, FRAHTOS (Proposed) has superior performance, enhancing efficiency from 88% with 5 nodes to 95% with 50 nodes. FRL occupies the second position with 93%. DDPG and AMOPG provide a relative enhancement, achieving 88% and 85% utility, respectively, but PFR-OA and GA-PSO exhibit inferior performance at 82% and 78% utility. This trend indicates that FRL algorithms, such as FRAHTOS and FRL, leverage network scalability more efficiently, as an increase in the number of nodes facilitates improved load distribution and concurrent task processing. The diminished enhancement of GA-PSO with the escalation of node quantity underscores its constraints in distributed IoT settings. This investigation affirms the pivotal importance of distributed architectures in enhancing efficiency and resource utilization.

The third graph in Fig. 4 illustrates that escalating task difficulty (low, medium, heavy) results in a reduction of the average utility of all algorithms, given a constant number of task (5000) and a constant number of nodes (20). Nonetheless, FRAHTOS (Proposed) exhibits optimal performance across all three difficulty levels, with its efficiency diminishing from 95% on light tasks to 88% on heavy tasks. Subsequently, FRL achieves an efficiency of 85% in demanding activities. Alternative algorithms, including DDPG, AMOPG, PFR-OA, and GA-PSO, attain efficiencies of 80%, 78%, 75%, and 70% on demanding tasks, respectively. The data indicate that FRAHTOS and FRL exhibit greater adaptability to complicated tasks owing to their three-tier designs and reinforcement learning algorithms, possibly attributable to their capacity for intelligent resource allocation and task-level optimization. The suboptimal performance of GA-PSO can be attributed to its generic approach to optimization problems, rendering it ineffective for the particular and real-time requirements of IoT. The reduction in utility with heightened complexity underscores that algorithms tailored for certain IoT settings can excel in demanding situations.

- ***Experiments 2***

**Fig. 5:**
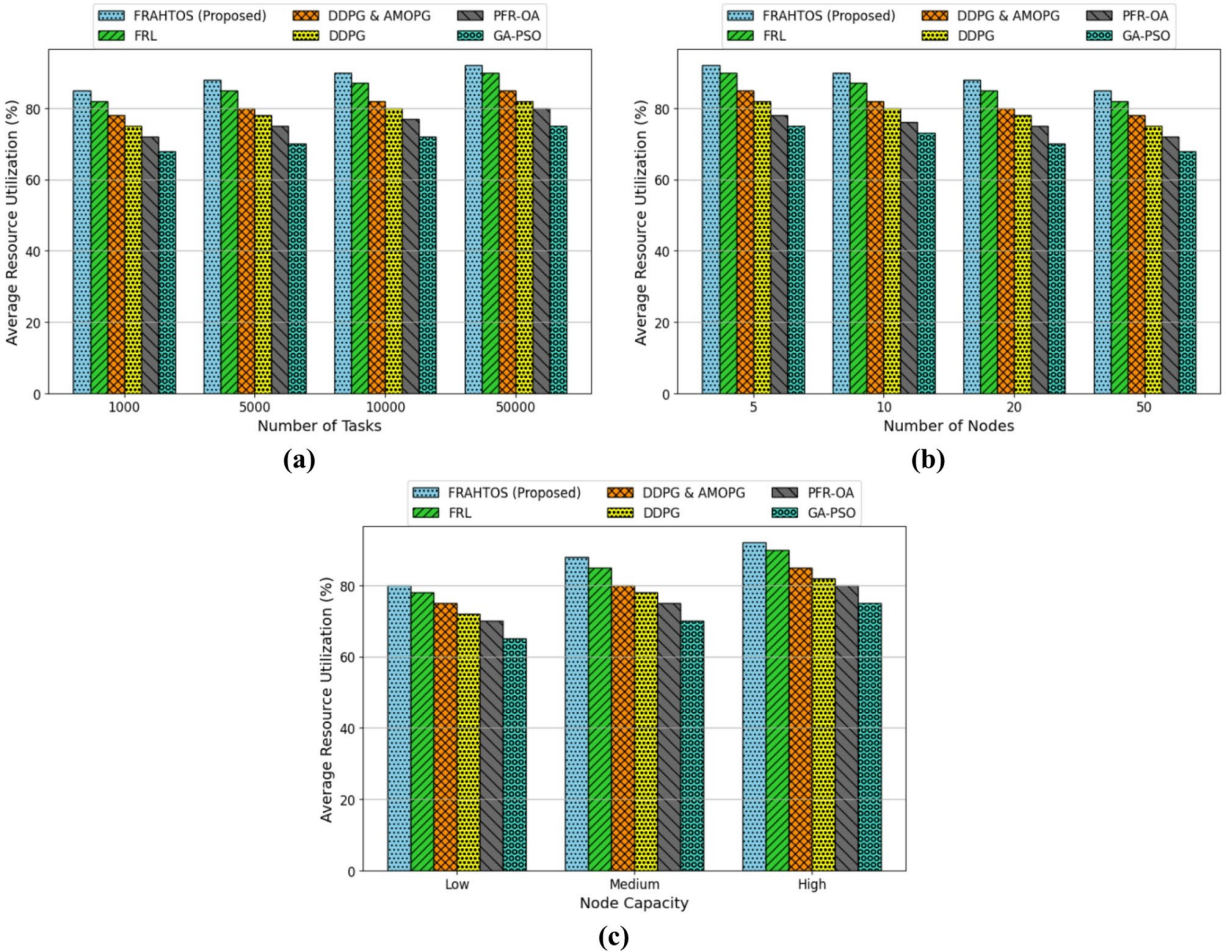
Comparison of mean resource consumption across distinct conditions: (**a**) fluctuating task quantities, (**b**) variable node quantities, and (**c**) differing node capacities. FRAHTOS exhibits superior resource management efficiency compared to baseline approaches.

The average resource consumption index has been examined as a crucial statistic for assessing the performance of algorithms in federated learning systems inside Internet of Things (IoT) contexts. This index reflects the efficiency of hardware resource use in network nodes for task processing. Three distinct scenarios, encompassing variations in work quantity, node count, and node capacity, have been analyzed to evaluate the performance of several algorithms, including the proposed FRAHTOS method, relative to previous benchmark techniques. The analysis findings are illustrated as bar graphs in Fig. 5.

The started graph in Fig. 5 illustrates the outcomes of resource consumption analysis in a scenario with varying task quantities (1000, 5000, 10000, 50000), while maintaining a constant number of nodes (20) and medium capacity. It demonstrates that the escalation of tasks from 1000 to 50,000 results in a progressive increase in resource utilization across all algorithms, signifying heightened strain on the computing infrastructure due to the augmented workload. The FRAHTOS (Proposed) algorithm has achieved superior resource usage, maintaining rates of 85% with 1000 tasks and 92% with 50,000 tasks. The FRL algorithm ranks second with a score of 90%. Conversely, the GA-PSO algorithm, exhibiting merely 75% efficiency under heavy load situations, reveals its constraints in addressing the scalability of IoT tasks. The superiority of FRAHTOS likely arises from its sophisticated resource allocation methods and adaptation to the 3-Tier design, resulting in enhanced performance in distributed environments. The results demonstrate that reinforcement learning algorithms, including FRAHTOS and FRL, exhibit superior stability and resource usage efficiency under escalating task loads.

The second graph in Fig. 5 which analyzes resource utilization by varying the number of nodes (5, 10, 20, 50) under a constant workload of 5000 tasks, indicates that an increase in the number of nodes correlates with a reduction in resource consumption across all algorithms. This reduction results from improved task distribution across additional nodes and less computing burden on each node. FRAHTOS demonstrated superior performance in this context, attaining an optimal equilibrium between efficiency and load distribution by decreasing resource use from 92% across 5 nodes to 85% across 50 nodes. The FRL algorithm exhibited satisfactory performance, achieving 82% accuracy with 50 nodes. Alternative algorithms, including DDPG, AMOPG, PFR-OA, and GA-PSO, demonstrated a more significant decrease in resource usage; nonetheless, their effectiveness remained inferior to that of FRAHTOS and FRL. These findings validate that employing distributed architectures, such as 3-Tier, alongside FRL techniques, can enhance resource efficiency and scalability.

The third graph in Fig. 5 analyzes the impact of varying node capacity (low, medium, high) under fixed conditions (5000 tasks and 20 nodes). The results demonstrate that augmenting the processing capacity of the nodes enhances the average resource usage across all algorithms, since high-capacity nodes facilitate the handling of additional workloads. In this context, FRAHTOS yields superior results, outperforming alternative approaches with utilization rates of 80%, 88%, and 92% for low, medium, and high capacities, respectively. The FRL algorithm is the nearest rival to FRAHTOS, achieving 90% efficiency at high capacity. Other algorithms exhibit far inferior performance at high capacity, particularly GA-PSO, which fails to exceed 75% even at maximum capacity. These results underscore that leveraging scalable architectures and adaptive resource allocation capabilities, exemplified by FRAHTOS, is crucial for improving performance across varying hardware conditions. Effective resource use in high-capacity nodes can serve as a crucial facilitator for the advancement of large-scale IoT infrastructures.

- ***Experiments 3***

**Fig. 6:**
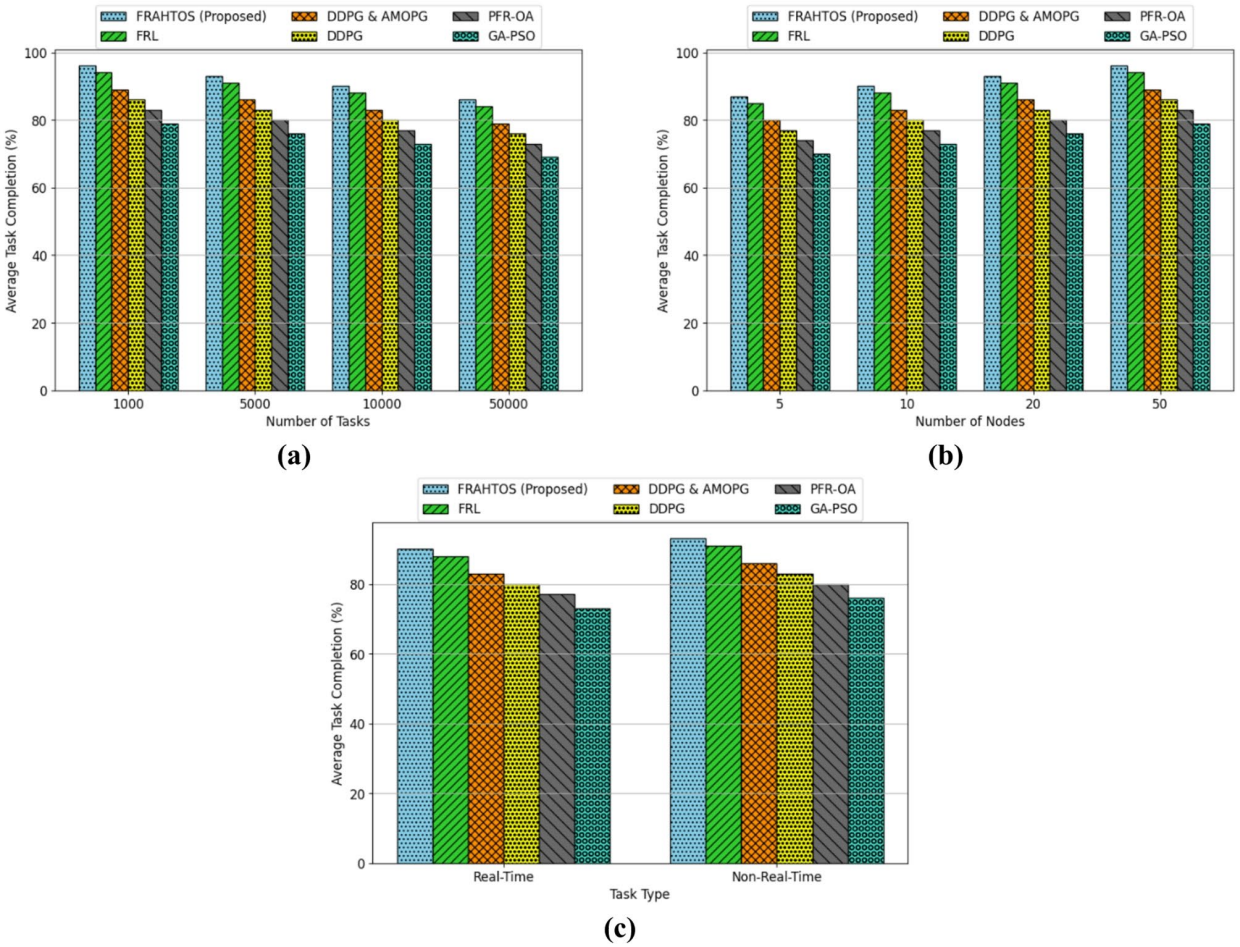
Comparison of task completion rates under several scenarios: (**a**) differing workload sizes, (**b**) varied numbers of nodes, and (**c**) real-time versus non-real-time workloads. FRAHTOS attains superior completion rates, particularly under extensive workloads.

The task completion rate is a crucial metric for assessing algorithm performance, reflecting the system’s capability to effectively execute designated tasks under varying situations. This signal gains significance particularly in situations involving substantial workload or time-critical tasks. This section analyzes three evaluation scenarios: variations in task quantity, computational node count, and task classification (real-time versus non-real-time). This investigation aims to compare the performance of the proposed FRAHTOS algorithm with reference algorithms FRL, DDPG, PFR-OA, and GA-PSO under diverse operating situations and to evaluate their adaptability to IoT processing requirements. The findings of this research are illustrated by graphs in Fig. 6.

An analysis of algorithm performance under escalating workload conditions indicates that as the number of tasks increases from 1,000 to 50,000 (within a 20-node environment and non-real-time workloads), all algorithms exhibit a relative decline in task completion rates. This reduction results from scalability issues and resource limitations when managing substantial workloads. The FRAHTOS (Proposed) algorithm has achieved a superior completion rate compared to alternative approaches, ranging from 96% in low-volume work to 86% in high-volume projects. The FRL algorithm ranks second with 84%, whilst GA-PSO has inferior performance at 69%. The results demonstrate that FRAHTOS exhibits superior scalability performance owing to its sophisticated resource allocation and task prioritization methods inside distributed architectures like 3-Tier. It further validates the superiority of reinforcement learning algorithms, including FRAHTOS and FRL, in handling substantial workloads in IoT situations.

The findings of the second analysis indicate that augmenting the number of nodes (from 5 to 50) in a scenario with 5000 non-real-time tasks resulted in a substantial enhancement of the task completion rate across all methods. This augmentation results from the enhancement in the allocation of computational load and the alleviation of stress on each node. In this situation, FRAHTOS demonstrates superior performance, elevating the completion rate from 87% across 5 nodes to 96% over 50 nodes. The FRL algorithm has a performance rate of 94% on 50 nodes. Conversely, GA-PSO, with a performance rate of 79%, exhibits constraints in leveraging distributed infrastructures. The findings suggest that federated algorithms utilizing reinforcement learning, such as FRAHTOS and FRL, derive greater advantages from structural scalability and can execute tasks in parallel more efficiently in environments with an increased number of nodes.

The third analytical scenario examines the impact of task type (real-time versus non-real-time) on the average completion rate, utilizing a fixed quantity of 5000 task and 20 nodes. The findings indicate that, overall, real-time activities exhibit a lower completion rate compared to non-real-time tasks, attributable to their heightened sensitivity to latency and the necessity for rapid responses. FRAHTOS delivers superior performance, with a 90% completion rate in real-time activities and a 93% completion rate in non-real-time tasks. FRL serves as the nearest competitor, with rates of 88% and 91%. Conversely, GA-PSO, exhibiting reduced rates particularly in real-time activities 73%, demonstrates insufficient adherence to the rapid processing demands in IoT. The results demonstrate that algorithms like FRAHTOS and FRL, which employ distributed architectures and targeted optimizations for time-sensitive contexts, possess a superior capacity to do tasks in real-time situations. Thus, a comprehensive analysis of energy use and the application of empirical data can delineate the trajectory for future enhancement.

- ***Experiments 4***

**Fig. 7:**
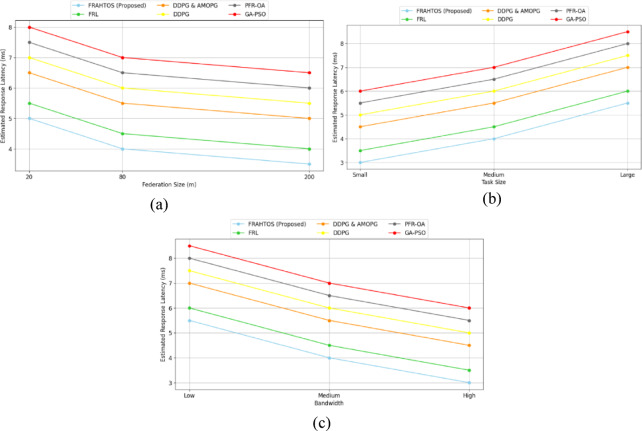
Anticipated response latency across varying operational parameters: (**a**) federation size, (**b**) task size, and (**c**) network bandwidth. FRAHTOS routinely attains reduced latency in comparison to rival methodologies.

Figure 7 presents a series of line graphs that evaluate the efficacy of scheduling algorithms in Internet of Things (IoT) contexts for projected response latency. This latency serves as a crucial metric for evaluating the quality of distributed and real-time processing in federated architectures. This investigation examines three critical operational variables federation size, task size, and network bandwidth to assess their influence on latency across various algorithms, in comparison to the suggested methodology. The results indicate that the FRAHTOS algorithm consistently yields the minimal reaction time across all cases. This benefit arises from its optimal design in dynamic task management and resource allocation inside three-layer distributed infrastructures.

The initial graph in Fig. 7 analyzes the impact of federation size (m = 20, 80, 200) on the predicted response latency in the context of medium-sized activities and moderate bandwidth. The findings indicate that an increase in the number of federation nodes correlates with a drop in latency across all algorithms; this decline is attributed to an improved distribution of computational load in larger networks. The proposed FRAHTOS algorithm demonstrates superior performance, achieving a latency reduction from 5.0 ms at m = 20 to 3.5 ms at m = 200, whereas FRL ranks second with a comparable reduction from 5.5 ms to 4.0 ms. Alternative algorithms, such as GA-PSO, exhibit inferior performance with latency varying from 8.0 to 6.5 ms. The statistics suggest that FRAHTOS exhibits significant scalability through the implementation of dynamic optimizations in resource allocation and task management within 3-Tier systems. Consequently, the reinforcement learning-based federated algorithms, particularly FRAHTOS and FRL, exhibit reduced latency in extensive IoT networks.

The second graph in Fig. 7 analyzes the impact of task size (small, medium, large) on the predicted response latency inside a network characterized by a constant federation size (m = 80) and medium bandwidth. As tasks expand, the computing demands escalate, resulting in increased latency for all methods. FRAHTOS (Proposed) demonstrates superior performance in this situation, with latency values of 3.0, 4.0, and 5.5 ms for small to big tasks. FRL functions as a direct competitor with a latency of 3.5 to 6.0 milliseconds. Conversely, algorithms like GA-PSO and PFR-OA attain latencies beyond 8 ms for substantial tasks. This performance disparity illustrates the superiority of federated algorithms grounded in reinforcement learning for managing substantial workloads inside distributed infrastructures. The targeted optimizations in FRAHTOS for resource allocation in high-load tasks render it an effective choice for managing complicated operations in IoT contexts.

The third graph in Fig. 7 examines the projected response latency over different network bandwidths (low, medium, high) while maintaining consistent task size and federation size (m = 80). The results indicate that augmenting the bandwidth substantially decreases latency across all methods, as data transfer velocity and inter-node coordination enhance. The proposed FRAHTOS algorithm demonstrated the lowest latency in this case, recording 5.5 ms at low bandwidth, 4.0 ms at medium bandwidth, and 3.0 ms at high bandwidth. FRL ranks second, with comparable latencies. Alternative algorithms, like GA-PSO and PFR-OA, exhibited inferior performance at low bandwidth, with latencies surpassing 8 ms. These findings underscore that using dispersed architectures and lowering communication overheads, as demonstrated in FRAHTOS, is crucial for minimizing latency in high-density networks. Consequently, enhancing communication efficiency under fluctuating bandwidth conditions is essential for the success of federated algorithms designed for IoT contexts.

- ***Experiments 5***

**Fig. 8:**
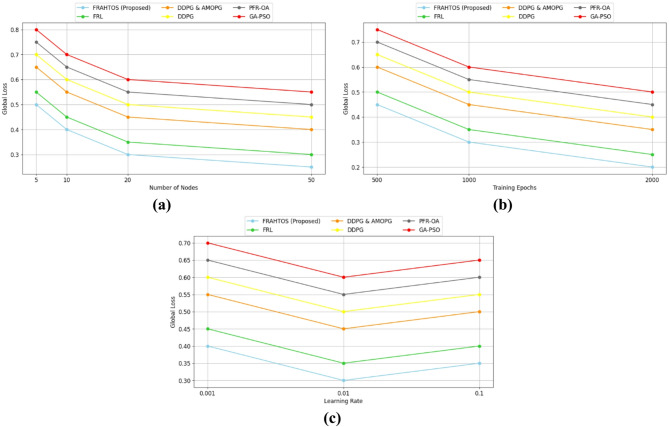
Trends in global loss convergence over several conditions: (**a**) differing node quantities, (**b**) variable training epochs, and (**c**) varying learning rates. FRAHTOS exhibits superior convergence speed and stability compared to baseline techniques.

The examination of global loss convergence in federated learning is crucial for assessing the stability and efficacy of the model training process. Figure 8 analyzes the global loss convergence trend concerning three principal variables: the number of nodes, the number of training cycles, and the learning rate for the FRAHTOS algorithm and other comparison methodologies. These assessments are performed to assess the stability, precision, and flexibility of the algorithms across various federated learning scenarios in IoT environments.

The initial line graph in Fig. 8 illustrates the global loss convergence trend for varying node quantities (5, 10, 20, 50) with a constant learning rate of 0.01 across 1000 training cycles. The results indicate that the suggested FRAHTOS algorithm consistently attains the lowest loss in comparison to the other algorithms evaluated. The augmentation of nodes results in a notable decrease in loss across all methodologies, attributable to the enhanced distribution of computational burden and the heightened involvement of nodes in the federated learning process. Specifically, FRAHTOS decreases the loss from 0.50 at 5 nodes to 0.25 at 50 nodes, and FRL declines from 0.55 to 0.30, securing second place. The enhancement in FRAHTOS performance is probably attributable to the implementation of efficient solutions in dynamic node management and model aggregation inside the 3-Tier hierarchical architecture. Conversely, the GA-PSO algorithm demonstrates inferior performance, with losses of 0.80 at 5 nodes and 0.55 at 50 nodes, highlighting its constraints in managing scalability within IoT systems. Collectively, our findings indicate that employing FRL methodologies at bigger scales can facilitate more rapid and efficient convergence.

The second graph in Fig. 8 analyzes the convergence trend of losses across varying training cycle counts (500, 1000, 2000), maintaining a constant number of nodes (20) and a learning rate (0.01). The findings indicate that augmenting the number of cycles resulted in a consistent decrease in losses across all algorithms, as additional time is allocated for optimizing the global model in the federated learning framework. The FRAHTOS algorithm demonstrates superior performance, achieving losses of 0.45, 0.30, and 0.20 at 500, 1000, and 2000 cycles, respectively. This is succeeded by FRL with losses of 0.50, 0.35, and 0.25. Alternative approaches, such as DDPG, AMOPG, PFR-OA, and GA-PSO, also exhibit decreases; nonetheless, they remain substantially inferior to the first two methods. The declining trend of loss in FRAHTOS is characterized by a steeper slope, signifying its substantial capacity to utilize extended durations for model enhancement. This advantageous performance likely arises from the algorithm’s tailored design for remote IoT contexts and the advantages of sophisticated parameter aggregation. Conversely, GA-PSO demonstrates comparatively diminished enhancement with an extended duration, attributable to its structural constraints on federated learning. This investigation reaffirms that algorithms tailored for federated contexts and IoT environmental conditions attain greater accuracy and stability in convergence over time.

The third panel of Fig. 8 analyzes the effect of learning rate on global loss convergence, presuming a fixed number of nodes (20) and training epochs (1000). The analysis of the findings indicates that a learning rate of 0.01 is the most optimal for all algorithms, as lower rates (0.001) result in sluggish learning progress, while higher rates (0.1) induce instability in convergence. The FRAHTOS algorithm surpasses all three-performance metrics, exhibiting losses of 0.40, 0.30, and 0.35 at rates of 0.001, 0.01, and 0.1, respectively, in contrast to FRL, which reported losses of 0.45, 0.35, and 0.40. Other algorithms, particularly GA-PSO, exhibit more losses at suboptimal learning rates, attributable to their inadequacy in effectively calibrating the learning parameters. The graph illustrates a U-shaped curve depicting the relationship between learning rate and loss rate, underscoring the necessity of identifying the ideal learning rate in distributed architectures. The superiority of FRAHTOS is due to its capacity to adaptively modify learning parameters and accommodate variations in learning rates within dynamic situations. These findings underscore the necessity for additional research utilizing authentic IoT data and incorporating supplementary criteria, such as the model’s long-term stability.

- ***Experiments 6***

**Fig. 9:**
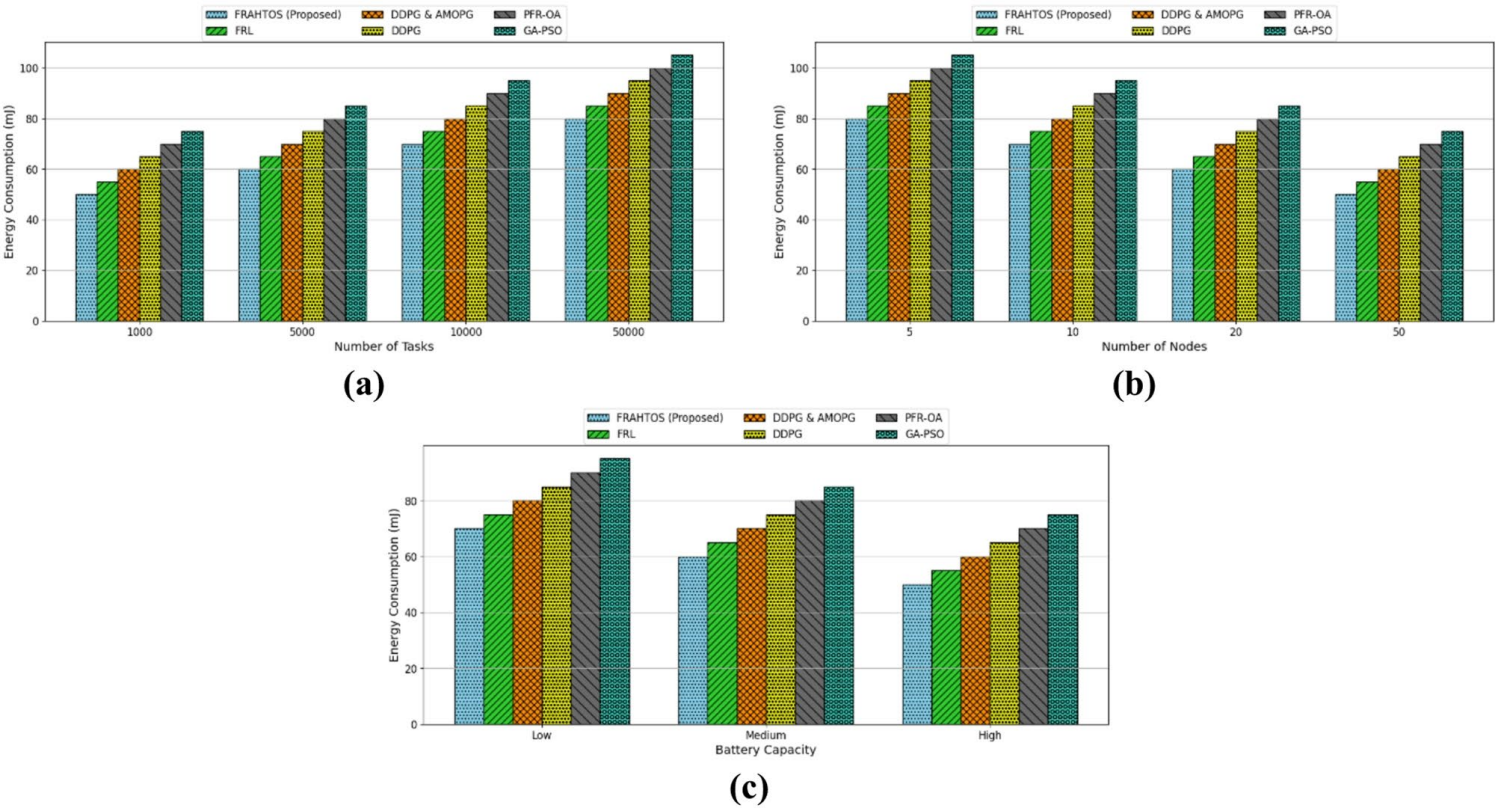
Energy consumption analysis under different conditions: (**a**) task volume, (**b**) node quantity, and (**c**) battery capacity levels. FRAHTOS exhibits markedly reduced energy consumption, particularly in resource-limited settings.

Figure 9 analyzes the energy consumption patterns of diverse algorithms across multiple federated learning situations inside IoT environments. Three principal metrics task quantity, node count, and battery capacity are regarded as independent variables to assess their influence on the algorithms’ energy efficiency. This investigation aims to assess the performance of the proposed FRAHTOS algorithm to other reference techniques under varying operating conditions and energy limitations in distributed systems.

The comparative energy consumption chart, featuring an escalating number of tasks (1000, 5000, 10000, 50000) with a constant node count (20) and average battery capacity, demonstrates that the FRAHTOS algorithm constantly surpasses the reference techniques. As the number of tasks rises, the energy consumption of all algorithms escalates, attributable to the augmented processing load. Nonetheless, FRAHTOS restricts energy usage to between 50 and 80 mJ, whereas FRL attains 85 mJ and GA-PSO achieves 105 mJ at 50,000 tasks. The disparity is ascribed to the optimization of task distribution and effective resource usage within the three-tier architecture. The boxplot distribution of the results indicates reduced dispersion and a lower energy median for FRAHTOS, suggesting its resilience under high load situations. These findings illustrate the superior capacity of FRAHTOS and FRL to manage substantial loads while optimizing energy consumption in IoT contexts.

An analysis of energy consumption across varying node counts (5, 10, 20, 50) with a constant task load (5,000) reveals that an increase in the number of nodes results in a reduction in energy consumption across all algorithms. This results from the distribution of the computational burden and the alleviation of stress on each node. FRAHTOS attains optimal performance by decreasing consumption from 80 mJ to 50 mJ, whilst FRL reduces consumption from 85 mJ to 55 mJ. Alternative approaches, like DDPG, PFR-OA, and GA-PSO, are constrained to an energy consumption of 60 to 75 mJ at 50 nodes, respectively. The diminutive boxes and reduced medians in FRAHTOS signify the steadiness of its energy usage. The decrease in energy usage with the addition of nodes substantiates the beneficial impact of scalability on enhancing energy efficiency, particularly in algorithms tailored for distributed architectures like FRAHTOS and FRL.

Analyzing energy expenditure across three battery capacity levels (low, medium, high) with a constant number of nodes (20) and tasks (5000) indicates that augmenting battery capacity results in decreased energy consumption across all methodologies. FRAHTOS utilizes 70, 60, and 50 mJ in low, medium, and high conditions, respectively, whereas FRL, its nearest competitor, consumes 75, 65, and 55 mJ. Algorithms like GA-PSO and PFR-OA exhibit deficiencies in responding to energy resource limitations, particularly at low capacities (up to 95 mJ), due to their high consumption rates. The box plot further demonstrates a more uniform distribution and less dispersion of FRAHTOS. The results demonstrate that FRAHTOS and FRL, through the implementation of adaptive energy management strategies, offer enhanced stability and efficiency in both energy-constrained and energy-abundant contexts.

**Table 3:** Performance comparison between FRAHTOS and baseline scheduling approaches in fog computing using comparative metrics.

Method	Utility (%)	Resource utilization (%)	Task completion (%)	Latency (ms)	Energy consumption (mJ)
FRAHTOS	85–95	85–92	86–96	3.0–5.5	50–80
FRL^38^	70–80	70–78	75–85	6.0–8.0	100–120
DDPG-AMOPG^6^	68–78	68–76	72–82	6.5–8.5	105–125
DDPG^16^	65–75	65–73	70–80	7.0–9.0	110–130
PFR-OA^23^	72–82	72–80	74–84	5.5–7.5	95–115
GA-PSO^37^	65–75	68–75	70–80	7.0–9.0	110–130

Table 3 presents a comparative analysis of the performance between FRAHTOS and Baseline task scheduling methodologies in fog computing. The table clearly demonstrates that FRAHTOS has superior performance, attaining minimal latency (3.0–5.5.0.5 ms), high efficiency (85–95%), and energy efficiency (50–80 mJ) in comparison to the reference baseline approaches.

Table 4 demonstrates that FRAHTOS substantially surpasses the most formidable competitor methodologies across all assessment metrics. The effect sizes are generally substantial (Hedges’ g > 0.9 or Cliff’s δ > 0.7), and all pairwise comparisons retain significance following multiple-comparison adjustment (adjusted *p* < 0.01).

**Table 4:** Statistical significance testing of FRAHTOS compared with baseline methods across all evaluation metrics (10 independent simulation runs, α = 0.05).

Metric	Test	Overall *p*	Primary contrast (FRAHTOS vs. best competitor)	Effect size (95% CI)	Adjusted *p*	Winner
Utility (%)	ANOVA + Tukey	< 0.001	FRAHTOS vs. FRL	Hedges’ g = 1.15 (0.78–1.49)	0.002	FRAHTOS
Resource Utilization (%)	Kruskal–Wallis + Dunn	< 0.001	FRAHTOS vs. FRL	Cliff’s δ = 0.72 (0.51–0.86)	0.004	FRAHTOS
Task Completion (%)	ANOVA + Tukey	< 0.001	FRAHTOS vs. FRL	Hedges’ g = 1.02 (0.70–1.35)	0.003	FRAHTOS
Latency (ms, lower = better)	Kruskal–Wallis + Dunn	< 0.001	FRAHTOS vs. FRL	Cliff’s δ = 0.80 (0.63–0.91)	0.001	FRAHTOS
Energy (mJ, lower = better)	ANOVA + Tukey	< 0.001	FRAHTOS vs. PFR-OA	Hedges’ g = 0.95 (0.62–1.28)	0.005	FRAHTOS

In addition to numerical comparisons, it is essential to examine the reasons for FRAHTOS’s persistent dominance in utility, latency, task completion, and energy efficiency. The enhancements arise from the synergistic functions of its elements. FRL is essential in minimizing latency, as dispersed agents acquire policies without the sharing of raw data, facilitating swifter decision-making and more consistent convergence compared to centralized DRL methods like DDPG or SAC. The incorporation of Earliest Deadline First (EDF) scheduling in FRL guarantees the prioritization of latency-sensitive tasks, a characteristic lacking in Greedy or Round-Robin benchmarks. The PSO-GA hybrid optimizer is essential for non-real-time workloads: PSO facilitates swift convergence, whereas GA promotes exploration diversity, thus mitigating the local optima issue inherent in PSO-exclusive approaches and the sluggish adaptation characteristic of GA-exclusive tactics. This synergy elucidates the observed increases in energy consumption, as workloads are assigned to suitable nodes, minimizing redundant computation.

The influence of VAE-based preprocessing and GMM/DBSCAN clustering is equally strong, markedly improving task grouping efficiency. The VAE minimizes computational and communication burdens by condensing high-dimensional IoT features into succinct latent representations, whilst the GMM offers probabilistic task allocations that adjust to fluctuations in workload. Competing FL-based frameworks like HAFedRL and PFR-OA, while excellent for privacy preservation, overlook clustering, resulting in elevated misallocation rates in varied environments. The integration of VARIMA forecasting enhances system stability by predicting workload variations and averting abrupt overloads an element often absents in standard methodologies.

### Constraints

Although FRAHTOS exhibits notable gains in latency reduction, energy efficiency, and scalability, it is imperative to recognize various limitations and hazards in order to maintain openness and academic integrity.

The incorporation of FRL, PSO–GA optimization, and VAE/GMM clustering inherently elevates the computing burden relative to more straightforward methods. While preprocessing with VAE decreases dimensionality, the training of federated models and hybrid optimization methods can be arduous, especially for extensive task sets.

Hardware limitations: Implementation on severely resource-limited edge devices continues to pose difficulties. FRAHTOS necessitates modest computational power and memory, perhaps surpassing the limitations of ultra-low-power IoT sensors. Practical solutions may necessitate heterogeneous hardware support or the offloading of specific components to fog nodes with more capacity.

Scalability in extensive federations: Although FRAHTOS is engineered to scale across diverse nodes, an increase in federated players to the thousands may result in synchronization delays, straggler effects, and elevated communication expenses. Hierarchical or asynchronous aggregation solutions may be necessary to address these challenges in practical situations.

Security and privacy vulnerabilities: Federated learning inherently minimizes raw data exchange; yet, it remains susceptible to security threats like poisoning attempts, model inversion, and the inference of sensitive task attributes. Although data-level privacy is maintained, supplementary measures like secure aggregation, differential privacy, or blockchain integration are essential to enhance security assurances.

## Conclusion

The proposed FRAHTOS framework demonstrates superior performance relative to benchmark algorithms by efficiently assigning tasks in fog computing settings and employing a sophisticated framework that integrates multiple unique methodologies. Experiments demonstrate that FRAHTOS continuously surpasses benchmarks in average utility (85% to 95%), average resource utilization (85% to 92%), and average task completion (86% to 96%) as the number of tasks, nodes, and task complexity increases. This advantage arises from dynamic resource management and effective task allocation within a 3-Tier architecture, which guarantees scalability and stability in IoT contexts. In the projected response latency benchmark (3.0 to 5.5 ms), FRAHTOS achieves the minimal delay using real-time clustering and EDF scheduling, essential for time-sensitive applications. At minimal energy consumption (50–80 mJ), the VAE and PSO-GA optimizations effectively reduce energy usage, which is essential for battery-limited nodes in IoT. At global convergence losses ranging from 0.20 to 0.50, FRAHTOS demonstrates expedited and more stable convergence through effective federal aggregation and dynamic learning rate adjustment.

Notwithstanding the encouraging efficacy of FRAHTOS in enhancing the federated learning process inside IoT networks, several significant difficulties impede its practical application. In high workload scenarios (e.g., 50,000 tasks), the substantial rise in energy consumption and the decline in total system efficiency from 95% to 85% signify scalability constraints in resource-limited nodes. Moreover, elevated learning rates (e.g., 0.1) induce instability in the global convergence of losses, underscoring the necessity for meticulous adjustment of learning parameters. These issues suggest that the implementation of FRAHTOS in actual IoT contexts may face impediments including hardware variety across nodes, uncertainty in network conditions, and limitations in resource allocation.

The simulation findings indicate that FRAHTOS has robust scalability, accommodating tens of thousands of tasks and hundreds of nodes; however, implementing the framework in extensive IoT ecosystems with numerous heterogeneous devices introduces further complexities. FRAHTOS utilizes FRL and distributed clustering to reduce communication cost and guarantee that model training and scheduling scale linearly with the number of nodes. The implementation of hybrid PSO–GA optimization and adaptive VAE compression diminishes computational demands, rendering the system viable for extensive networks. Nonetheless, practical implementation may face obstacles including stragglers and unstable nodes during federated updates, non-stationary traffic patterns that diminish clustering precision, and synchronization expenses in extensive federated learning. These concerns indicate that although FRAHTOS offers a scalable framework, additional research incorporating hierarchical federated models, asynchronous aggregation, and real-world deployment validations will be crucial to verify scalability in practical IoT contexts.

Future research should concentrate on enhancing clustering methods and reinforcement learning to address these constraints. Utilizing authentic IoT data rather of simulated data (such as iFogSim) can yield a more precise evaluation of FRAHTOS performance under practical situations, particularly regarding energy consumption and system stability. Moreover, integrating federated learning simulators like TensorFlow Federated (TFF) with iFogSim can yield a more comprehensive examination of model convergence and system performance across varying scales. Enhancing the DQN agent with deeper neural networks and implementing dynamic adaptive clustering can augment the algorithm’s scalability and efficiency in more intricate IoT contexts.

## Data Availability

The datasets used and/or analyzed during the current study available from the corresponding author on reasonable request. The code and materials used to generate the results in this manuscript have been deposited on Zenodo and are publicly available at: 10.5281/zenodo.17041525 The record includes source code and accompanying files; use is subject to the license specified on the Zenodo record. No additional restrictions apply. Further details are available from the corresponding author upon reasonable request.
